# Mutation patterns in recurrent and/or metastatic oropharyngeal squamous cell carcinomas in relation to human papillomavirus status

**DOI:** 10.1002/cam4.3741

**Published:** 2021-02-01

**Authors:** Henrike Reder, Steffen Wagner, Nora Wuerdemann, Christine Langer, Sarah Sandmann, Andreas Braeuninger, Martin Dugas, Stefan Gattenloehner, Claus Wittekindt, Jens Peter Klussmann

**Affiliations:** ^1^ Department of Otorhinolaryngology, Head and Neck Surgery Medical Faculty Justus‐Liebig University Giessen Giessen Germany; ^2^ Department of Otorhinolaryngology, Head and Neck Surgery Medical Faculty University Hospital Cologne Cologne Germany; ^3^ Center for Molecular Medicine Cologne (CMMC) University of Cologne Faculty of Medicine University Hospital Cologne Cologne Germany; ^4^ Institute of Medical Informatics Westphalian Wilhelms University Muenster Muenster Germany; ^5^ Department of Pathology Medical Faculty Justus‐Liebig University Giessen Giessen Germany

**Keywords:** human papillomavirus, next‐generation sequencing, oropharyngeal cancer, p53 gene, recurrence, squamous cell carcinoma

## Abstract

Patients with HPV‐driven (HPV+) oropharyngeal squamous cell carcinoma (OPSCC) have a significantly improved overall survival compared to patients with HPV‐negative (HPV−) OPSCC. Nevertheless, 13%–25% of patients with HPV+OPSCC develop local/distant recurrence (LDR) and have a course of disease similar to HPV−OPSCC. We hypothesize that HPV+OPSCCs of patients with LDR have a mutation frequency and pattern similar to HPV−OPSCCs, which is associated with severe outcome. We performed targeted next‐generation sequencing using a customized gene panel and compared data from 56 matched HPV+and HPV−OPSCC of patients with/without LDR regarding protein‐altering variants. Despite improved overall survival of patients with HPV+OPSCC, those who develop LDR show a strongly reduced survival rate that is similar or even worse compared to HPV−OPSCC patients. Overall, the number of mutations was similar in OPSCC of patients with and without LDR. In total and with respect to *TP53*, HPV−OPSCC had significantly more protein‐altering mutations than HPV+OPSCC. The number of mutations was similar in HPV−OPSCC of patients with and without LDR with the exception of *FAT1*, which was mutated more frequently in patients without LDR. In HPV+OPSCC, *HRAS*, *PIK3R1*, *STK11* and *TP63* were more frequently mutated in patients with LDR compared to patients without. HPV+OPSCC of patients with LDR have a similar mutation pattern as HPV−OPSCC, except *TP53*, which was mutated to a significantly lower extent. In conclusion, HPV−and HPV+OPSCC with LDR have similar mutation counts in the analyzed genes. We suspect that the number of mutations is not causal for disease progression, rather specific mutations could be important.

## Graphical abstract

A subgroup of 13–25% of HPV‐driven (HPV+) OPSCC patients develop local or distant recurrence (LDR) and a severe course of disease similar to that of patients with HPV‐negative (HPV‐) OPSCC. Regarding the genes analyzed, we found that the HPV+OPSCC of patients with LDR carried a higher number of mutations than HPV+OPSCC of patients without LDR, with a similar mutation frequency as the HPV−OPSCC of LDR patients. Surprisingly, HPV−OPSCC of patients who did not develop LDR showed the highest number of mutations, suggesting that not the number but specific driver mutations are dominant factors for recurrence and that tumor‐specific neoantigens may play a role in a supportive antitumor immune response after therapy.

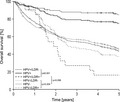

## 1 INTRODUCTION

In 2018, approximately 92,900 new cases of oropharyngeal squamous cell carcinoma (OPSCC) were diagnosed worldwide and a high mortality rate of about 50% is reported.^1^ In contrast to other head and neck cancers, the incidence of OPSCC is increasing and in many countries a growing number of cases are attributed to high‐risk human papillomavirus (HPV).^2^, ^3^, ^4^ These patients are characterized by a specific risk profile, comparatively good response to therapy, and superior overall survival (OS) rates.^5^, ^6^ Consequently, strategies to de‐escalate the treatment of patients with HPV‐driven (HPV+) OPSCC are investigated. Notably, two clinical trials investigating de‐escalating therapy found no benefit in replacing cisplatin as standard of care in radio‐chemotherapy by the epidermal growth factor inhibitor cetuximab. In addition, treatment with cetuximab resulted in inferior overall and progression‐free survival compared to treatment with cisplatin.^7^, ^8^

It is known that the HPV early proteins E6 and E7 have a transforming potential.^9^ Briefly, E7 binds the active retinoblastoma protein (Rb), thereby releasing the transcription factor E2F, which drives the cell cycle into S‐phase. E6 interacts with a ubiquitin ligase that labels the tumor suppressor p53 for proteolytic degradation,^10^, ^11^, ^12^ thereby inhibiting cell cycle control and apoptosis. Consequently, HPV+tumors are not dependent on *TP53* mutations as a prerequisite for oncogenesis, and *TP53* mutations are rare in this entity. On the other hand, HPV−OPSCC often carry *TP53* variants.^13^, ^14^

Despite the generally good response to therapy and the good prognosis, approximately 13–25% of patients with HPV+OPSCC develop local or distant recurrence (LDR) within 2 years after current standard treatment^15^, ^16^ and a correspondingly unfavorable course of the disease. In a previous study, we demonstrated that HPV+OPSCC patients with LDR had a higher number of mutations than HPV+OPSCC patients without LDR. In particular, the frequency of mutations in *HRAS*, *PIK3R1*, *STK11*, and *TP63* was elevated.^17^ However, genetic differences between HPV−and HPV+OPSCC of patients with and without LDR still need to be elucidated.

We hypothesize that mutation numbers and patterns in OPSCC are similar in patients with LDR irrespective of HPV status. Therefore, we determined the total number of LDR in patients diagnosed with HPV+and HPV−OPSCC at our hospital between 2000 and 2018. We matched respective patients with HPV−OPSCC with/without LDR to cases of a previously analyzed cohort with HPV+OPSCC and compared frequency and patterns of protein‐altering mutations by targeted next‐generation sequencing using a customized gene panel.

## 2 MATERIALS AND METHODS

### 2.1 Patient selection

We retrospectively analyzed the HPV and LDR status of all patients with a primary OPSCC diagnosed at our hospital between 2000 and 2018. For the targeted next‐generation sequencing (targeted NGS) and survival analysis, we excluded those patients diagnosed after November 2014 in the group without LDR to ensure an event‐free follow‐up of at least 5 years. HPV association (HPV+) was defined by overexpression of CDKN2A (p16^INK4A^) and detection of high‐risk HPV‐DNA as previously described.^18^, ^19^, ^20^, ^21^ HPV+OPSCC were all positive for high‐risk HPV‐type 16 (*n* = 139) except two cases (one was positive for type 58, and the other for types 35 and 26), which were not included in our analysis. Among all patients with HPV+OPSCC, 21 (15.1%) patients were identified who developed LDR. Of those, primary tumor tissue meeting our inclusion criteria for sequencing analysis (see below) was available for 14 cases. Patients with established distant metastases at time of primary diagnosis were excluded from the targeted NGS analysis. DNA quality and/or quantity of FFPE tissue samples of five patients were insufficient for sequencing. To each of the remaining patients, we assigned corresponding cases with HPV+OPSCC without LDR and patients with HPV−OPSCC with and without LDR according to N‐stage, risk factors (nicotine/alcohol consumption and age), T‐stage, and gender (Table 1 and Table S1). Finally, a balanced cohort of 56 OPSCC with 14 cases in each group (HPV+/‐, with/without LDR) was analyzed by targeted NGS. A schematic representation of the study design is shown in Figure S1. Twelve HPV+OPSCC, each of patients with and without LDR, were investigated previously and results from targeted NGS analyses have been published before.^17^ All patients were treated by surgery with or without adjuvant radio‐/chemoradiotherapy, or by definitive radio‐/chemoradiotherapy following approved guidelines. Patients gave written informed consent and the study was approved by the local ethics committee.

**TABLE 1:** Characteristics of OPSCC and patients with and without LDR according to HPV status included in the targeted NGS analysis (*n* = 56).

		HPV+(*n* = 28)		HPV−(*n* = 28)		Matching priority
		LDR+ (*n* = 14)	LDR− (*n* = 14)	LDR+ (*n* = 14)	LDR− (*n* = 14)	
Age (years)	Median	65.8	57.4	67.2	67.1	**6**
	Mean	66.8	59.5	61.8	62.8	
		*n* (%)	*n* (%)	*n* (%)	*n* (%)	
Age group	<60 years	6 (42.9)	9 (64.3)	6 (42.9)	5 (35.7)	
	≥60 years	8 (57.1)	5 (35.7)	8 (57.1)	9 (64.3)	
Gender	Female	4 (28.6)	4 (28.6)	1 (7.1)	2 (14.3)	**5**
	Male	10 (71.4)	10 (71.4)	13 (92.9)	12 (85.7)	
T‐stage	T1	2 (14.3)	3 (21.4)	2 (14.3)	2 (14.3)	**4**
	T2	2 (14.3)	6 (42.9)	6 (42.9)	6 (42.8)	
	T3	6 (42.8)	2 (14.3)	3 (21.4)	4 (28.6)	
	T4	4 (28.6)	3 (21.4)	3 (21.4)	2 (14.3)	
N‐stage	N0	1 (7.1)	1 (7.1)	2 (14.3)	1 (7.1)	**1**
	N+	13 (92.9)	13 (92.9)	12 (85.7)	13 (92.9)	
M‐stage	M0	7 (50.0)	14 (100.0)	9 (64.3)	14 (100.0)	
	M+	7 (50.0)	0 (0.0)	5 (35.7)	0 (0.0)	
Smoking	Yes	9 (64.3)	8 (57.1)	11 (78.6)	9 (64.3)	**2**
	No	5 (35.7)	6 (42.9)	3 (21.4)	5 (35.7)	
Alcohol	Yes	2 (14.3)	1 (7.1)	7 (50.0)	4 (30.8)	**3**
	No	12 (85.7)	13 (92.9)	7 (50.0)	9 (69.2)	
	Unknown	—	—	—	1	
Surgery	Yes	6 (42.9)	11 (78.6)	7 (50.0)	9 (64.3)	**—**
	No	8 (57.1)	3 (21.4)	7 (50.0)	5 (35.7)	
Radiotherapy	Yes	12 (85.7)	12 (92.3)	12 (85.7)	14 (100.0)	**—**
	No	2 (14.3)	1 (7.7)	2 (14.3)	0 (0.0)	
	Unknown	—	1	—	—	
Chemotherapy	Yes	11 (78.6)	7 (50.0)	9 (64.3)	6 (42.9)	**—**
	No	3 (21.4)	7 (50.0)	5 (35.7)	8 (57.1)	

### 2.2 DNA isolation and sequencing library preparation

DNA isolation and library preparation were performed as described previously.^17^ Briefly, DNA was isolated from macrodissected formalin‐fixed paraffin‐embedded (FFPE) primary tumor tissue with the Maxwell^®^ 16 FFPE Plus LEV DNA purification kit (Promega) following manufacturer's protocol. DNA quantity was determined with the Qubit™ 1.0 fluorometer (Thermo Fisher Scientific, Agilent Technologies) and the High Sensitivity DNA Kit (Agilent 2100 Bioanalyzer). Subsequently, 10 ng DNA per sample was prepared following the protocol for the Ion AmpliSeq™ DNA and RNA Library Preparation kit (Thermo Fisher Scientific). Sequencing was performed on the Ion Torrent PGM (Thermo Fisher Scientific).

### 2.3 Targeted NGS panel and variant calling

DNA sequencing with customized gene panel and variant calling has been described previously.^17^ In brief, the targeted NGS panel comprises the coding sequence of *TP53* (exon 2–exon 11) and 90 target regions in 22 genes (Table S2), which were frequently mutated in HPV+OPSCC.^14^, ^22^, ^23^, ^24^, ^25^, ^26^, ^27^ Primers were designed with the Ion AmpliSeq Designer v5.4.2 (Thermo Fisher Scientific). Data were analyzed with appreci8^28^ and aligned to the reference genome GRCh37 (hg19). Minimum read depth was set to 50, minimum number of variant allele reads to 20, the alternative's average base quality of reads to at least 15, the difference of the average base quality between reference and alternative must not be greater than 7, and the variant allele frequency had to be at least 5%. Only protein‐altering mutations were considered for further analysis.

### 2.4 Statistical analysis

Analyses were performed using the SPSS statistical software (IBM SPSS 26.0). Overall survival (OS) was calculated from date of initial diagnosis (date of routine biopsy confirmed by histology) to the date of death due to any cause or to the last known date when the patient was still alive. OS rates were calculated by the Kaplan‐Meier method and the significance of differences was calculated by log‐rank test. Qualitative and quantitative data between all cohorts were compared using Kruskal–Wallis test, and between distinct cohorts using two‐sided Mann–Whitney *U* test for independent samples. Significance of results was expected for *p*‐values ≤0.05.

## 3 RESULTS

### 3.1 OS and occurrence of LDR in relation to HPV status

For the OS and targeted NGS analysis, we included patients with LDR diagnosed up to 2018 to increase the number of samples with the restriction that patients without LDR were only included with the primary diagnosis before November 2014 to ensure event‐free follow‐up for at least 5 years. In this patient cohort, 23.2% were tested positive for p16^INK4A^ and high‐risk HPV‐DNA; 15.1% of those patients developed LDR within 5 years after treatment. In contrast, patients with HPV−OPSCC developed LDR in 28.0% of cases (*p* = 0.002, Table 2).

**TABLE 2:** Five‐year survival, recurrence, and mutation numbers in patients with OPSCC stratified according to HPV and LDR status

	HPV+(*n* = 139)		HPV−(*n* = 461)		*p*‐value
Recurrence rate [%]	15.1		28.0		**0.002** ^a^
of those distant metastasis [%]	71.4		55.0		
of those local recurrence [%]	28.6		45.0		
5y‐OS [%]	75.3		43.6		**≤0.001** ^b^ (Figure 1)

	HPV+(*n* = 28)		HPV−(*n* = 28)		*p*‐value
Total number of mutations in the analyzed genes (mean)	183 (6.5)		464 (16.6)		**0.002** (Figure 2A)
Number of *TP53* mutations (mean)	27 (1.0)		95 (3.4)		**≤0.001** (Figure 2A)

	HPV+/ LDR+ (*n* = 14)	HPV+/ LDR− (*n* = 14)	HPV‐ / LDR+ (*n* = 14)	HPV‐ / LDR− (*n* = 14)	*p*‐value
5y‐OS [%]	16.2	85.1	37.3	45.9	
Patients with mutations (%)	8 (57.1)	5 (35.7)	12 (85.7)	13 (92.9)	**0.004** ^c^
Total number of mutations in the analyzed genes (mean)	165 (11.8)	18 (1.3)	129 (9.2)	335 (23.9)	**0.005** ^c^
Patients with *TP53* mutations (%)	5 (35.7)	2 (14.3)	12 (85.7)	12 (85.7)	**≤0.001** ^c^
Number of *TP53* mutations (mean)	25.(1.8)	2 (0.1)	31 (2.2)	64 (4.6)	**≤0.001** ^c^

	LDR+ (*n* = 28)		LDR− (*n* = 28)		*p*‐value
Total number of mutations in the analyzed genes (mean)	294 (10.5)		353 (12.6)		0.540 (Figure 2B)
Number of *TP53* mutations (mean)	56 (2.0)		66 (2.4)		0.510 (Figure 2B)

*p*‐values (asymptotic, two‐sided) calculated by Mann–Whitney *U* test.

Abbreviations: HPV−, HPV‐negative OPSCC; HPV+, HPV‐driven OPSCC; LDR−, patients without local or distant recurrence; LDR+, patients with local or distant recurrence.

^a^ Pearson's Chi‐square test.

^b^ Kaplan‐Meier, log‐rank test.

^c^ Kruskal‐Wallis test; bold: *p*‐values ≤0.05.

**FIGURE 1:**
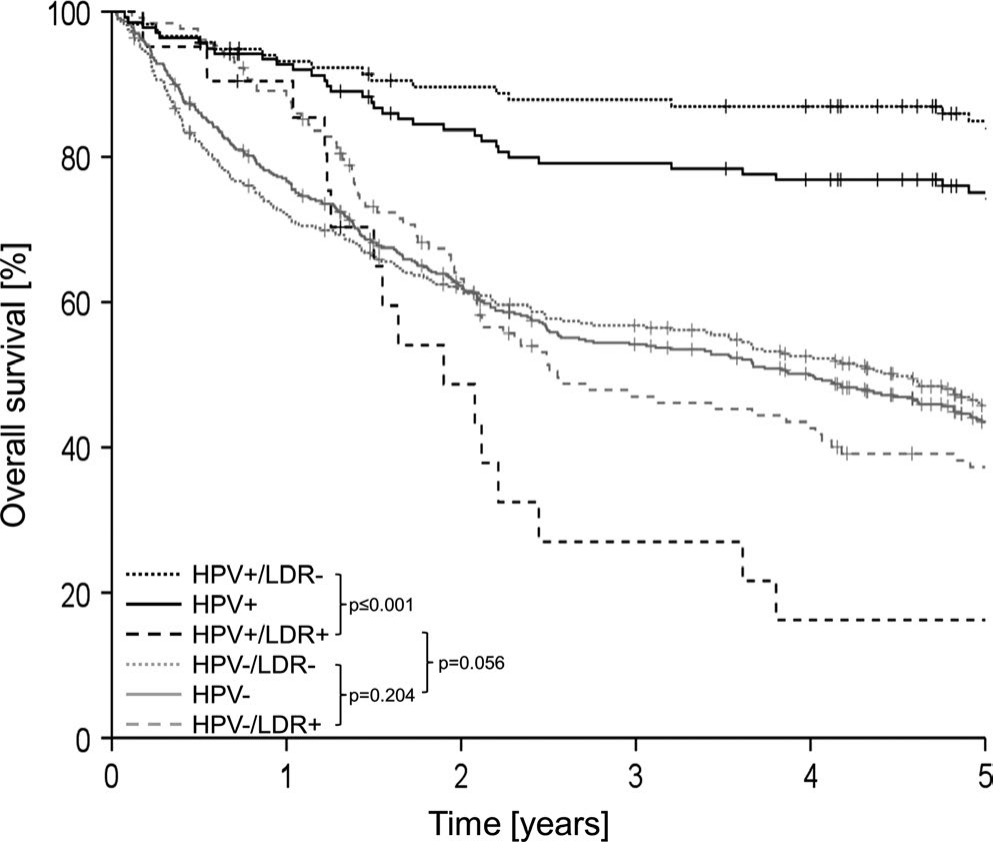
Influence of local/distant recurrence (LDR) on overall survival of patients with HPV−(*n* = 461, gray) and HPV+(*n* = 139, black) OPSCC. Dashed lines: patients with LDR (HPV+: *n* = 21; HPV−: *n* = 129); dotted lines: patients without LDR (HPV+: *n* = 118; HPV−: *n* = 332). Censored patients (alive at last follow‐up) are indicated as vertical markers. *p*‐values were calculated by log‐rank test

The OS of patients by HPV and LDR status is shown in Figure 1. Patients with HPV+OPSCC have a significantly improved OS compared to patients with HPV−OPSCC (5y‐OS: 75.3% vs. 43.6%). Divided into subgroups, HPV+OPSCC patients without LDR showed the most favorable OS rate with a 5‐year OS of 85.1%. Regarding patients with HPV−OPSCC, those without LDR showed improved OS compared to patients with LDR without reaching statistical significance (5y‐OS: 45.9% vs. 37.3%; *p* = 0.204). Patients with LDR and HPV+OPSCC have a substantially reduced OS (5y‐OS: 16.2%), which is even worse than that of patients with HPV−OPSCC independent of LDR (*p* = 0.056).

### 3.2 Targeted NGS of the HPV−OPSCC cohort

**FIGURE 2:**
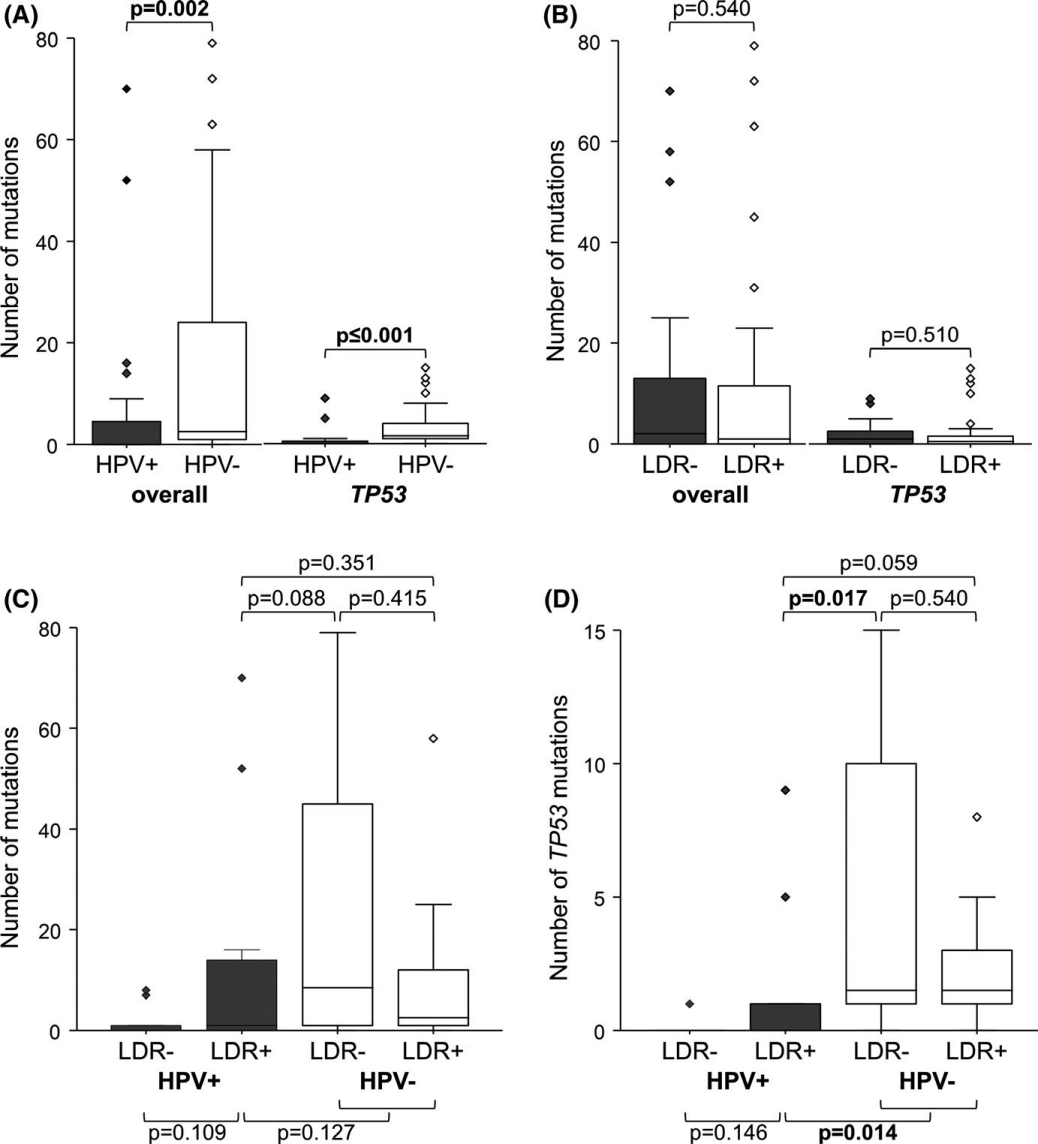
Number of mutations detected by targeted NGS in all genes studied and in TP53 in HPV+and HPV−OPSCC of patients with and without local/distant recurrence. Overall and *TP53* mutation numbers of (A) HPV−and HPV+OPSCC (*n* = 28, each) and (B) OPSCC in patients with and without LDR (*n* = 28, each). Overall (C) and *TP53* (D) mutation numbers of analyzed OPSCC subgroups stratified by LDR and HPV status (*n* = 14, each). Center lines show the medians; box limits indicate the 25th and 75th percentiles; whiskers extend 1.5 times the interquartile range from the 25th and 75th percentiles; outliers are represented by dots. *p*‐values were calculated by Mann–Whitney *U* test for independent samples; *p*‐values ≤0.05 in bold

Primary tumor tissue of matched patients with HPV−OPSCC was sequenced with a custom targeted NGS panel and data were compared to previously analyzed HPV+OPSCC (Figure 2). Only protein‐altering mutations were considered in the analyses. OPSCC cohort characteristics, matching criteria, and patient characteristics are listed in Table 1 and Tables S1 and S3. The average base coverage of HPV−OPSCC in targeted NGS was 4214 reads and 98% of bases in the sequenced region had more than 100 reads (in comparison to an average base coverage of 3289 reads and 97% of bases with more than 100 read of the previously reported sequencing of HPV+OPSCC^17^). Regarding *TP53*, the average base coverage was 3328 reads, with 2999 reads in HPV+and 3659 reads in HPV−OPSCC. Each gene was mutated in at least one primary tumor of both HPV−OPSCC subgroups with and without LDR. In HPV−OPSCC, tumors of patients with LDR harbored 129 mutations (mean: 9.2, median: 2.5 mutations/tumor) altogether, while tumors of patients without LDR harbored 335 mutations (mean: 23.9 mutations/tumor; median: 8.5 mutations/tumor; *p* = 0.415, Mann‐Whitney U test, Figure 2C). *TP53* was by far the most frequently mutated gene in HPV−OPSCC patients (85.7%) irrespective of the LDR status. No *TP53* mutation was detected in 2/14 (14.3%) patients in each HPV−OPSCC subgroup. HPV−OPSCC of patients without LDR had altogether 64 *TP53* mutations compared to 31 variants in HPV−OPSCC in patients with LDR; however, the median number of *TP53* mutations per tumor is similar and the comparison did not reach significance (*p* = 0.540; Figure 2D). The next most frequently mutated genes in HPV−OPSCC of patients with LDR were *TP63*, *PIK3CA*, and *DDX3X* (each mutated in 4/14 tissue samples, 28.6%). In HPV−OPSCC of patients without LDR, the most frequent mutated genes were *TP63* and *FAT1* with aberrations in seven samples (50.0%) each. The individual mutation counts of the listed genes between patients with and without LDR of HPV−OPSCC were similar (Table S4). Only *FAT1* was mutated at a significantly higher frequency in patients without LDR than in patients with LDR (*p* = 0.043; Table S4). Altogether 28 base variants resulted in truncated proteins in seven patients without LDR, while in five LDR patients nine variants led to truncated proteins. Fourteen and 18 variants resulted in a frameshift in the primary tumor tissue of patients with and without LDR, respectively. All variants of each patient are listed in Table S5.

### 3.3 Number of mutations in the genes studied and in *TP53* is not a predictor for recurrence status

The visualization of mutation numbers in HPV+and HPV−OPSCC by LDR status is shown in Figure 2 and summarized in Table 2. In ascending order, the fewest mutations were found in HPV+OPSCC of patients without LDR, followed by HPV−and HPV+OPSCC, each of patients with LDR; the most mutations were found in HPV−OPSCC of patients without LDR (Figure 2A). Regardless of HPV status, we found no difference in total and *TP53* mutation counts between patients with and without LDR (Figure 2B). After classification into the four subgroups (HPV−/HPV+, with/without LDR), there is no significant difference in the total number of mutations neither within HPV+(*p* = 0.109) nor within HPV−(*p* = 0.415) OPSCC (Figure 2C). In patients with LDR, the mutation number of HPV+OPSCC is similar to that of HPV−OPSCC (*p* = 0.351). HPV−OPSCC of patients without LDR presented a higher number of mutations than HPV+OPSCC of patients with LDR (*p* = 0.088; Figure 2C). Within the HPV−OPSCC subgroup, there is no difference in the number of *TP53* mutations (*p* = 0.540; Figure 2D). However, HPV+OPSCC of patients with LDR had less mutations in *TP53* compared to HPV−OPSCC in general (*p* = 0.014; Figure 2D) or compared to subgroups stratified by LDR status (LDR+: *p* = 0.059; LDR‐: *p* = 0.017; Figure 2D). The mutation numbers altogether and with respect to *TP53* differ significantly between the four subgroups (Table 2).

### 3.4 Comparison of mutation patterns in HPV+and HPV−OPSCC of patients with/without LDR

**FIGURE 3:**
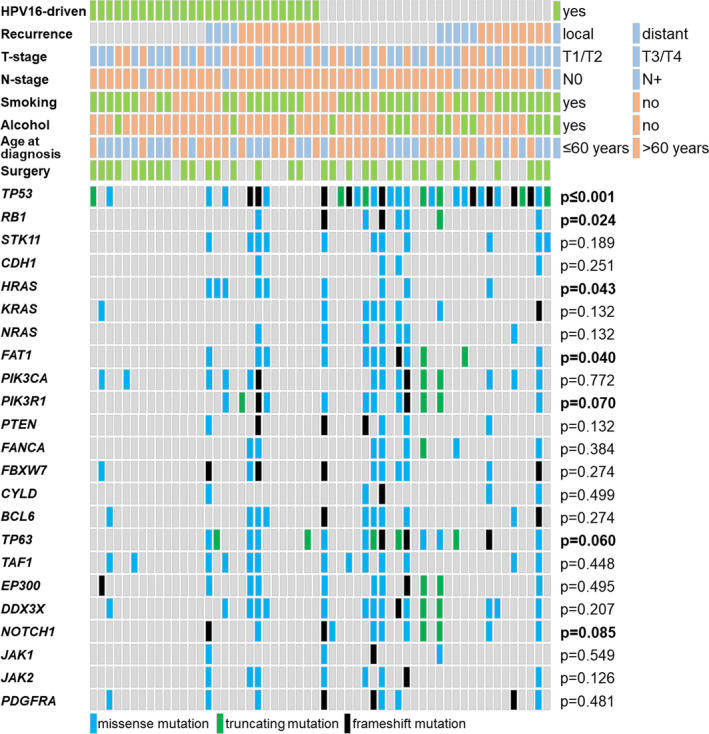
OPSCC and patient characteristics in comparison to gene mutations. Patients are displayed in columns (*n* = 56) in the group with HPV+(left) and HPV−(right) OPSCC. Each row corresponds to OPSCC and patient characteristics (top), or to one or more regions of the indicated genes, which were analyzed by targeted NGS (bottom). Smoking: yes: ≥10 pack‐years, no: <10 pack‐years; Alcohol: yes: ≥2 standard glasses/day, no: <2 standard glasses/day; gray: characteristic not applicable or no mutation detected; blue: missense mutation; black: truncating mutation; green: frameshift mutation. *p*‐values were calculated by Kruskal‐Wallis test for independent samples; *p*‐values ≤0.1 in bold

By comparing mutations in HPV+and HPV−OPSCC, some differences become visible; besides significantly more *TP53* mutations in HPV−OPSCC (*p* ≤ 0.001), further differences in the mutation number were found in *FAT1* (*p* = 0.096), *KRAS* (*p* = 0.045), *NOTCH1* (*p* = 0.071), *NRAS* (*p* = 0.045), and *RB1* (*p* = 0.045). Divided into the four subgroups, we observed that HPV+OPSCC of patients without LDR had the least number of mutations, followed by HPV−and HPV+OPSCC of patients with LDR, whereas the highest number of mutations was found in HPV−OPSCC of patients without LDR (Figure 2C). Comparing HPV+OPSCC of patients with LDR with HPV−OPSCC (with or without LDR), *TP53* is the only gene with a significant difference; it is less frequently mutated in HPV+OPSCC of patients with LDR (5/14, 36%) compared to HPV−OPSCC with LDR (12/14, 86%, *p* = 0.059) and HPV−OPSCC without LDR (12/14, 86%, *p* = 0.017) (Table S6). Significantly different mutation frequencies between all four subgroups were found in *TP53* (*p* ≤ 0.001), *RB1* (*p* = 0.024), *HRAS* (*p* = 0.043), and *FAT1* (*p* = 0.040), and trending toward significance were *PIK3R1* (*p* = 0.070), *TP63* (*p* = 0.060), and *NOTCH1* (*p* = 0.085) (Figure 3). Compared to the other subgroups, HPV−OPSCC of patients without LDR had the highest number of mutations in *RB1, KRAS, NRAS, FAT1, PIK3R1, PTEN, TP63, and NOTCH1*; HPV+OPSCC of patients with LDR had highest mutation frequencies in *HRAS* (Figure 3).

## 4 DISCUSSION

We observed tremendous differences in OS rates not only between patients with HPV+and HPV−OPSCC, but especially with regard to recurrence status (Figure 1). We hypothesized that mutation frequencies and pattern in OPSCC are similar in patients with LDR regardless of HPV status and we assumed that gene mutations are an important driver for LDR. Therefore, we investigated HPV−OPSCC of patients with and without LDR by targeted NGS and compared frequencies and patterns of protein‐altering mutations to previously analyzed primary tumors of HPV+OPSCC.^17^

The OS rate is significantly worse in patients with HPV+OPSCC and LDR compared to patients with HPV+OPSCC without LDR and surprisingly, even compared to patients with HPV−OPSCC. In contrast, there is no significant difference in OS of HPV−OPSCC patients with and without LDR. These observations are in contrast to the current literature, which reports that recurred p16‐positive OPSCC patients survived significantly longer than their p16‐negative counterparts.^16^, ^29^ This might be due to significant differences in treatment regimens (Table S3).^30^ Patients with HPV+OPSCC and LDR received less frequent radiotherapy compared to patients with HPV−OPSCC with LDR with a trend toward significance (*p* = 0.079, Mann–Whitney *U* test; data not shown). Additionally, age at primary diagnosis could play a role in this context since the HPV+OPSCC patients with LDR were older than patients in the other groups (Table S3). The higher age we observed in patients with HPV+compared to HPV−OPSCC is in contrast to the general opinion that patients with HPV+OPSCC are younger. Nevertheless, as we mentioned in the recent publication,^6^ most currently published data are based on clinical trials with selected patient cohorts or registry data without the inclusion of experimental data on HPV status. An important aspect for OS in patients with LDR is the rate of distant metastasis, which is 50% in HPV+compared to 17.3% in HPV−OPSCC (Table S3). The rate of distant metastasis in HPV−OPSCC is consistent with the literature,^31^ while different rates are described for HPV+head and neck cancer, ranging from similar ^32^ to higher rates ^33^ in HPV+compared to HPV−cases. However, the observed high rate of distant metastasis in patients with LDR and HPV+OPSCC could also contribute to a worse survival compared to the other subgroups.

In a previous study, we investigated HPV+OPSCC and observed severely reduced survival rates of patients with LDR. Our study revealed that patients with LDR had higher mutation counts detected by targeted NGS using a custom gene panel and especially oncogenes and transcription factors were affected.^17^ Subsequently, the question raised whether similar or different mutations are found in HPV−OPSCC of patients with LDR. In line with previous studies, we observed higher mutation numbers in HPV−tumors compared to HPV+tumors.^34^, ^35^, ^36^ This is not surprising given the underlying carcinogenic mechanisms. Nevertheless, other studies found no difference in the number of mutations between HPV+and HPV−OPSCC.^14^, ^22^ Therefore, additional data are required, especially considering the low number of HPV+cancers and the inhomogeneous cancer entities in previous studies.

Regardless of HPV, we could not find a significant difference between patients with and without LDR in terms of the total or *TP53* mutation numbers (Figure 2B). Furthermore, there is no difference in the number of total and *TP53* mutations, neither within HPV+nor within HPV−OPSCC stratified by LDR status (Figure 2C and D). We detected a higher *TP53* mutation frequency in HPV−OPSCC than in HPV+OPSCC (*p* ≤ 0.001; Figure 2D), which is consistent with the literature.^13^, ^34^, ^37^ Therefore, *TP53* variants do not appear to influence the course of disease in HPV−OPSCC, but it remains open whether *TP53* mutations play a role in the development of recurrence in HPV+OPSCC.

With 7/28 (25.0%) cases, we detected higher *TP53* mutation frequencies in HPV+OPSCC than previously described. In HPV+cancers, malignant cells arise primarily from the degradation of p53 and deregulation of Rb by viral E6 and E7 protein activity; however, not necessarily from the accumulation of mutations in these genes. Therefore, *TP53* is usually present in its wild‐type form. For example, one study detected *TP53* mutations in 84.8% of HPV−head and neck squamous cell carcinoma (HNSCC), but only in 2.8% HPV+HNSCC.^14^ However, a similar molecular profile was described previously, including an enriched *TP53* mutation frequency of 3/20 (15%) HPV+OPSCC from patients with LDR.^38^ The even higher frequency of *TP53* mutations in our study could be a consequence of the composition of the cohort, that is, the enrichment of patients with LDR who have an increased number of *TP53* mutations (Figure 3).

Mutations in the gene FAT atypical cadherin 1 (*FAT1*) were elevated in HPV−OPSCC of patients without LDR compared to HPV−OPSCC of patients with LDR (*p* = 0.094). The human *FAT1* regulates cell growth, migration, and polarization with a dual role as tumor suppressor and oncogene.^39^ In esophageal SCC, *FAT1 *has been reported to inhibit tumor growth and epithelial–mesenchymal transition,^40^ which is an important step during metastasis. Another study found a strong association between *FAT1* mutations and an improved OS in patients with HPV−but not in HPV+HNSCC patients.^41^ In line with this, we detected a higher number of mutations in HPV−OPSCC of patients without LDR than in HPV−OPSCC with LDR (22 vs. 5 variants). Our results support the hypothesis that mutations in *FAT1* negatively affect tumor progression in HPV−OPSCC patients.

Regarding the mutation frequency within all four subgroups, differences in statistical comparison seem to result from the high mutation frequency in HPV−OPSCC of patients with LDR and the very low mutation frequency in HPV+OPSCC of patients without LDR. The significant differences in OS and our sequencing data suggest that recurrence in HPV−OPSCC and HPV+OPSCC is caused by different mechanisms. Specific mutations in HPV−OPSCC could drive the tumor into progression and hematogenic and lymphogenic spread. On the other hand, a higher *TP53* mutation frequency in addition to specific driver mutations could promote the development of recurrence in HPV+OPSCC.

The low number of patients with HPV+OPSCC and LDR is a weakening of our study. Although the incidence of HPV+OPSCC is increasing, the total number of patients developing LDR is rare. Therefore, we were restricted to a retrospective study design and the use of archived FFPE samples. We also used targeted NGS and did not apply whole exome (WES) or whole genome sequencing, which requires high‐quality DNA. As above, this is an unavoidable drawback related to the type of available samples. Hence, our data revealed interesting information, but underline the fact that prospective multicenter studies are needed, including frozen tissue biobanking and in‐depth analysis of gene variants in these clinically relevant subtypes of OPSCC. WES was applied in a previous study showing that metachronous HPV‐related OPSCC shares a genomic landscape with HPV‐unrelated cancers and that HPV‐related OPSCC which recurred is genetically similar to non‐recurring HPV‐related OPSCC.^36^ These findings are consistent with our results. Nevertheless, and in contrast to our study, the data derived from available WES datasets ^14^ and own sequencing data (using WES and targeted NGS to an unspecified extend) represent different tumor localization and were based on p16^INK4A^ staining without detection of HPV‐DNA in some samples, which is not sufficient for reliable HPV categorization.^42^, ^43^

In summary, we found that HPV+OPSCC of patients with LDR carried a higher number of mutations in the analyzed genes than HPV+OPSCC of patients without LDR, with a mutation frequency similar to that of HPV−OPSCC of LDR patients. Surprisingly, the primary tumor tissue of patients with HPV−OPSCC who did not develop LDR had the highest number of mutations. Furthermore, *TP53* variants do not seem to influence the course of disease in HPV−OPSCC. However, their role for development of recurrence in HPV+OPSCC remains open. Although our study is focused on protein‐altering variant in a selected number of genes, one could hypothesize that specific driver mutations, rather than the number of mutations, are dominant factors in recurrence. Additional whole‐genome‐based studies are needed to investigate this speculation. In addition, it can be speculated that a higher number of tumor‐specific neoantigens is caused by a higher number of mutations in HPV−OPSCC.^44^ In conclusion, this could be a reason for an antitumor immune response after therapy that supports a favorable course of disease in these cases. This is in contrast to cases with fewer mutations, which as a consequence develop LDR more frequently.

## CONFLICT OF INTEREST

All authors declare that they have no competing interests.

## ETHICAL CONSIDERATION

The study was approved by the local ethics committee (reference number: 95/15).

## Supporting information

Figure S1

Table S1

Table S2

Table S3

Table S4

Table S5

Table S6

## Data Availability

The datasets used and analyzed during the current study are available from the corresponding author on reasonable request.
