# Supplementary material for: Mutation patterns in recurrent and/or metastatic oropharyngeal squamous cell carcinomas in relation to human papillomavirus status
Source: Cancer Med. 2021 Feb 1;10(4):1347–56. doi: 10.1002/cam4.3741 (PMC7926014; doi:10.1002/cam4.3741)
Supplement: Supplementary file 1 — Figure S1 [file CAM4-10-1347-s001.pdf]

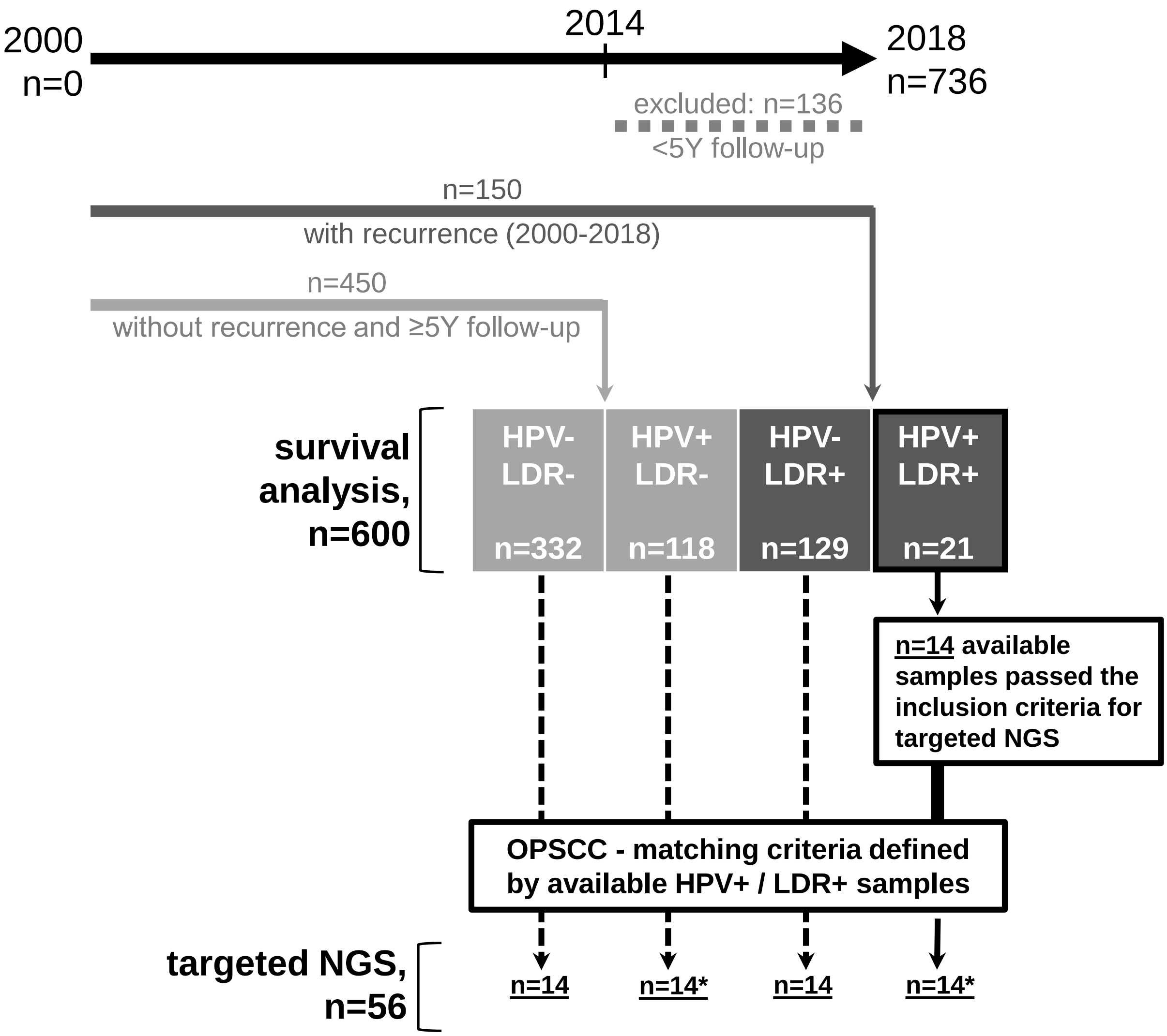

Supplemental Figure 1: Schematic representation of the study design. Among all patients with a primary OPSCC diagnosed at our hospital and HPV-status determined by CDKN2A (p16INK4A) immunohistochemistry and detection of high-risk HPV-DNA, we included all patients who developed local/distant recurrence (LDR) and, in the group without LDR, only patients diagnosed before November 2014 to ensure an event-free follow-up of at least five years. The patient group with HPV+OPSCC and LDR+ was used to define criteria to select the best-matched patients from the remaining groups for targeted NGS analysis. * n=12/14 samples have been analyzed in a previous study [17. Reder H, Wagner S, Gamerdinger U, Sandmann S, Wuerdemann N, Braeuninger A, et al. Genetic alterations in human papillomavirus-associated oropharyngeal squamous cell carcinoma of patients with treatment failure. Oral Oncol. 2019;93:59-65.]
