# Supplementary material for: Mutation patterns in recurrent and/or metastatic oropharyngeal squamous cell carcinomas in relation to human papillomavirus status
Source: Cancer Med. 2021 Feb 1;10(4):1347–56. doi: 10.1002/cam4.3741 (PMC7926014; doi:10.1002/cam4.3741)
Supplement: Supplementary file 2 — Table S1 [file CAM4-10-1347-s002.pdf]

Supplementary Table 1: Matching characteristics of the sequencing cohort (n=56).

Matching priority	Matching pair number	01				02				03				04			
	HPV16-driven	1	0	0	1	0	0	1	1	1	1	0	0	1	0	1	0
	LDR	1	1	0	0	0	1	1	0	1	0	0	1	1	1	0	0
	Failure type (local/distant)	D	L	0	0	0	L	L	0	D	0	0	D	D	D	0	0
4	T-stage	2	2	2	2	1	2	1	1	3	2	3	4a	4b	4b	2	2
1	N-stage	1	1	1	1	2a	2c	2b	2a	2c	2a	2c	2b	2c	2c	2c	2c
	M-stage	0	0	0	0	0	0	1	0	1	0	0	1	1	1	0	0
2	Smoking (≥10 pack years)	1	0	1	1	0	1	0	0	1	1	1	1	1	1	1	1
3	Alcohol (≥2 standard glasses)	0	1	0	0	0	1	0	0	0	0	0	0	0	0	0	1
5	Gender	M	M	F	F	M	M	M	M	M	F	F	M	M	M	M	M
6	Age	58.7	64.4	67.4	66.0	59.2	70.5	68.3	70.7	63.4	49.2	66.6	71.0	79.3	52.2	57.0	49.7
	surgery	0	0	0	1	1	1	1	1	0	1	0	0	0	0	1	1
	Radiotherapy	1	1	1	1	1	1	0	0	1	1	1	0	1	1	1	1
	Chemotherapy	1	1	1	0	0	0	0	0	1	0	1	0	1	1	0	1

Supplementary Table 1: continued

Matching priority	Matching pair number	05				06				07				08			
	HPV16-driven	0	1	1	0	1	0	1	0	0	1	0	1	1	0	1	0
	LDR	0	0	1	1	0	0	1	1	0	0	1	1	0	0	1	1
	Failure type (local/distant)	0	0	D	D	0	0	L	L	0	0	L	L	0	0	D	D
4	T-stage	3	1	4b	4b	3	2	3	2	2	4a	3	3	4	4b	4b	2
1	N-stage	2b	2b	2b	2b	0	0	0	0	2b	2b	2b	2b	2b	2b	2b	2b
	M-stage	0	0	1	0	0	0	0	0	0	0	0	0	0	0	0	1
2	Smoking (≥10 pack years)	1	1	1	1	0	0	1	1	1	1	1	1	1	1	1	1
3	Alcohol (≥2 standard glasses)	0	0	0	0	0	0	0	0	1	1	1	1	0	1	1	1
5	Gender	M	M	M	M	M	M	M	M	M	F	M	M	M	M	M	M
6	Age	67.7	59.1	60.0	51.6	78.3	84.4	81.1	51.6	44.4	57.9	67.4	57.6	53.8	46.3	70.6	64.1
	surgery	1	1	0	0	0	1	0	1	1	1	1	1	0	0	1	1
	Radiotherapy	1	1	1	1	0	1	1	0	1	1	1	1	1	1	1	1
	Chemotherapy	0	0	1	1	1	0	1	0	1	1	0	1	1	1	1	1

Supplementary Table 1: continued

Matching priority	Matching pair number	09				10				11				12			
	**HPV16-driven**	0	1	0	1	1	0	1	0	1	0	0	1	0	1	0	1
	**LDR**	1	0	0	1	0	1	1	0	0	1	0	1	1	0	0	1
	**Failure type (local/distant)**	D	0	0	D	0	D	D	0	0	D	0	L	D	0	0	D
4	**T-stage**	3	4a	4b	4	1	1	3	2	2	2	3	2	2	2	2	3
1	**N-stage**	2b	2b	1	2	1	0	1	1	2b	2b	2b	2b	1	2b	2b	2c
	**M-stage**	0	0	0	1	0	0	0	0	0	0	0	0	1	0	0	1
2	**Smoking (≥10 pack years)**	1	0	0	0	1	1	1	1	0	0	1	0	1	1	1	1
3	**Alcohol (≥2 standard glasses)**	0	0	1	0	0	1	0	0	0	0	0	0	0	0	0	0
5	**Gender**	M	F	M	F	M	F	F	M	M	M	M	M	M	M	M	0
6	**Age**	51.6	76.4	66.8	83.6	48.8	55.3	43.0	46.6	48.1	68.4	69.0	91.8	71.2	48.4	38.9	48.3
	**surgery**	0	0	1	0	1	1	1	1	1	0	0	1	1	1	1	0
	**Radiotherapy**	1	1	1	0	1	1	1	1	1	1	1	1	1	?	1	1
	**Chemotherapy**	1	1	1	0	1	0	1	0	1	1	0	0	1	0	0	1

Supplementary Table 1: continued

Matching priority	Matching pair number	13				14			
	**HPV16-driven**	1	0	0	1	0	1	0	1
	**LDR**	0	1	0	1	0	0	1	1
	**Failure type (local/distant)**	0	L	0	D	0	0	D	D
4	**T-stage**	2	3	1	0	3	3	1	3
1	**N-stage**	2b	2b	2a	2b	2b	2b	2b	2c
	**M-stage**	0	0	0	1	0	0	1	0
2	**Smoking (≥10 pack years)**	0	0	0	0	0	0	1	0
3	**Alcohol (≥2 standard glasses)**	0	1	0	0	?	0	1	0
5	**Gender**	M	M	M	F	M	M	M	M
6	**Age**	54.0	70.2	71.5	57.7	71.5	64.9	55.4	72.2
	**surgery**	1	1	1	1	0	0	1	0
	**Radiotherapy**	1	1	1	1	1	1	1	1
	**Chemotherapy**	1	1	1	1	1	1	1	1

L: local recurrence; D: distant recurrence; ?: information missing; 1: yes (except for TNM-stage); 0: no (except for TNM-stage); M: male; F: female
