# Supplementary material for: Mutation patterns in recurrent and/or metastatic oropharyngeal squamous cell carcinomas in relation to human papillomavirus status
Source: Cancer Med. 2021 Feb 1;10(4):1347–56. doi: 10.1002/cam4.3741 (PMC7926014; doi:10.1002/cam4.3741)
Supplement: Supplementary file 3 — Table S2 [file CAM4-10-1347-s003.pdf]

Supplementary Table 2: List of all gene regions covered by the targeted next generation sequencing panel.

Gene	Chr	HGVS nomenclature	Bases covered	Reference
BCL6	3	NM_001130845<br>ENST00000232014	187447070-187447195<br>187447290-187447419	[14]<br>[14]
CDH1	16	NM_004360<br>ENST00000261769	68846084-68846210<br>68847181-68847266	[14]<br>[25]
CYLD	16	NM_015247<br>ENST00000311559	50788250-50788329<br>50811765-50811882<br>50818250-50818321<br>50821628-50821738	[14]<br>[14]<br>[14]<br>[14]
DDX3X	X	NM_001193416<br>ENST00000399959	41193510-41193635<br>41200798-41200922<br>41201945-41202060<br>41202947-41203059<br>41206128-41206250	[22]<br>[22]<br>[22]<br>[22]<br>[14]
EP300	22	NM_001429<br>ENST00000263253	41545024-41545135<br>41553348-41553465<br>41565444-41565564<br>41565554-41565644<br>41566427-41566526	[22]<br>[14]<br>[14, 22]<br>[22]<br>[14, 22]
FANCA	16	NM_000135<br>ENST00000389301	89849224-89849351<br>89858341-89858427<br>89877157-89877269	[14]<br>[14]<br>[14]
FAT1	4	NM_005245<br>ENST00000441802	187540286-187540407<br>187630380-187630501<br>187630491-187630607	[22]<br>[22]<br>[22]
FBXW7	4	NM_033632<br>ENST00000281708	153247255-153247377<br>153249362-153249483	[22, 23]<br>[22, 23]
HRAS	11	NM_176795<br>ENST00000311189	533483-533599<br>533782-533882<br>534220-534306	[22]<br>[22, 24]<br>[24]
JAK1	1	NM_002227<br>ENST00000342505	65330444-65330567	[23]
JAK2	9	NM_004972<br>ENST00000381652	5050868-5050959<br>5064908-5065027<br>5066684-5066784	[22]<br>[22]<br>[22]
KRAS	12	NM_033360<br>ENST00000256078	25398189-25398310	[22]
NOTCH1	9	NM_017617<br>ENST00000277541	139402686-139402815<br>139412222-139412322<br>139413077-139413166<br>139417508-139417632	[14]<br>[14]<br>[14]<br>[22]
NRAS	1	NM_002524<br>ENST00000369535	115256463-115256578	[22]
PDGFRA	4	NM_006206<br>ENST00000257290	55140978-55141092<br>55151525-55151647	[14]<br>[22]
PIK3CA	3	NM_006218	178916618-17891674	[22]

		ENST00000263967	178916694-178916785<br>178916808-178916900<br>178916890-178917005<br>178936020-178936128<br>178936908-178937021<br>178938806-178938928<br>178952019-178952135	[22]<br>[14]<br>[22]<br>[14, 22-24]<br>[14]<br>[22]<br>[14, 22]
PIK3R1	5	NM_181523<br>ENST00000521381	67576309-67576424<br>67588070-67588192<br>67589569-67589628<br>67591063-67591180<br>67594360-67594440	[14]<br>[26]<br>[26]<br>[22]<br>[22]
PTEN	10	NM_000314<br>ENST00000371953	89692775-89692895<br>89717693-89717772	[14]<br>[22, 23]
RB1	13	NM_000321<br>ENST00000267163	48936961-48937044<br>48937034-48937120<br>49027106-49027221<br>49033881-49034001	[22]<br>[22]<br>[22, 23]
STK11	19	NM_000455<br>ENST00000326873	1220384-1220502<br>1221220-1221332	[14]<br>[14]
TAF1	X	NM_004606<br>ENST00000373790	70612739-70612851<br>70683754-70683840	[14]<br>[27]
TP53	17	NM_000546<br>ENST00000269305	7572848-7572976<br>7572966-7573067<br>7573878-7574004<br>7573995-7574070<br>7576495-7576597<br>7576579-7576702<br>7576830-7576949<br>7576947-7577059<br>7577032-7577141<br>7577120-7577220<br>7577393-7577511<br>7577509-7577613<br>7578209-7578294<br>7578305-7578439<br>7578431-7578560<br>7578096-7578219<br>7579277-7579391<br>7579390-7579515<br>7579509-7579588<br>7579576-7579684<br>7579728-7579850<br>7579840-7579960	
TP63	3	NM_001114978<br>ENST00000264731	189456393-189456511<br>189526221-189526350<br>189585582-189585702<br>189586378-189586459<br>189586449-189586531<br>189587088-189587171<br>189608593-189608722	[24]<br>[24]<br>[22]<br>[22]<br>[22]<br>[22]<br>[22, 24]
