# Supplementary material for: Mutation patterns in recurrent and/or metastatic oropharyngeal squamous cell carcinomas in relation to human papillomavirus status
Source: Cancer Med. 2021 Feb 1;10(4):1347–56. doi: 10.1002/cam4.3741 (PMC7926014; doi:10.1002/cam4.3741)
Supplement: Supplementary file 4 — Table S3 [file CAM4-10-1347-s004.pdf]

Supplementary Table 3: Tumor- and patient characteristics of 600 primary OPSCC patients analyzed for overall survival.

Risk factors		all (n=600)	HPV+		HPV-		p-value
			LDR+ (n=21)	LDR- (n=118)	LDR+ (n=129)	LDR- (n=332)	
		n (%)	n (%)	n (%)	n (%)	n (%)	
**Gender**	**male**	462 (77.0)	15 (71.4)	84 (71.2)	113 (87.6)	250 (75.3)	**0.010**
	**female**	138 (23.0)	6 (28.6)	34 (28.8)	16 (12.4)	82 (24.7)	
**Age**	**Median (years)**	60.3	70.6	61.0	58.8	60.3	**≤0.001#**
**IQR**	**1st quartile (years)**	53.5	58.2	52.8	52.0	54.1	
	**3rd quartile (years)**	67.3	79.5	68.4	65.8	66.7	
**Alcohol**	**>2 standard drinks/day**	304 (55.8)	3 (16.7)	20 (18.7)	81 (67.5)	200 (66.7)	**≤0.001**
	**≤2 standard drinks/day**	241 (44.2)	15 (83.3)	87 (81.3)	39 (32.5)	100 (33.3)	
	**unknown**	55	3	11	9	32	
**Smoking**	**>2 pack years**	481 (81.8)	11 (55.0)	64 (55.2)	115 (90.6)	291 (89.5)	**≤0.001**
	**≤2 pack years**	107 (18.2)	9 (45.0)	52 (44.8)	12 (9.4)	34 (10.5)	
	**unknown**	12	1	2	2	7	
**Packyears (smoker)**	**Median**	38,0	31,0	22,8	40,0	40,0	**≤0.001§**
	**1st quartile (years)**	25,0	22,5	10,0	30,0	27,0	
	**3rd quartile (years)**	50,0	58,5	40,0	58,0	51,0	
	**smokers without packyear specified (n)**	89	4	12	20	53	

Supplementary Table 3: continued

**UICC 8**	**I-III**	285 (47.9)	8 (38.1)	114 (96.6)	39 (30.2)	124 (37.9)	**≤0.001**
	**>III**	310 (52.1)	13 (61.9)	4 (3.4)	90 (69.8)	203 (62.1)	
	**I**	120 (20.2)	2 (9.5)	61 (51.7)	11 (8.5)	46 (14.1)	**≤0.001¶**
	**II**	82 (13.8)	3 (14.3)	32 (27.1)	14 (10.9)	33 (10.1)	
	**III**	83 (13.9)	3 (14.3)	21 (17.8)	14 (10.9)	45 (13.8)	
	**IV-IVa**	213 (35.8)	12 (57.1)	4 (3.4)	59 (45.7)	138 (42.2)	
	**IVb**	74 (12.4)	0 (0.0)	0 (0.0)	17 (13.2)	57 (17.4)	
	**IVc**	23 (3.9)	1 (4.8)	0 (0.0)	14 (10.9)	8 (2.4)	
	**unknown**	5	0	0	0	5	
**T-stage**	**1-2**	307 (51.5)	8 (38.1)	83 (70.3)	65 (50.4)	151 (46.0)	**≤0.001**
	**3-4**	289 (48.5)	13 (61.9)	35 (29.7)	64 (49.6)	177 (54.0)	
	**1**	139 (23.3)	3 (14.3)	37 (31.4)	18 (14.0)	81 (24.7)	**≤0.001¶**
	**2**	169 (28.4)	5 (23.8)	46 (39.0)	47 (36.4)	71 (21.6)	
	**3**	120 (20.1)	8 (38.1)	14 (11.9)	28 (21.7)	70 (21.3)	
	**4**	30 (5.0)	1 (4.8)	5 (4.2)	6 (4.7)	18 (5.5)	
	**4a**	57 (9.6)	0 (0.0)	11 (9.3)	12 (9.3)	34 (10.4)	
	**4b**	81 (13.6)	4 (19.0)	5 (4.2)	18 (14.0)	54 (16.5)	
	**unknown**	4	0	0	0	4	
**N-stage**	**N0**	166 (28.0)	18 (85.7)	15 (12.7)	34 (26.8)	114 (35.0)	**≤0.001**
	**N+**	426 (72.0)	3 (14.3)	103 (87.3)	93 (73.2)	212 (65.0)	
	**N0**	166 (28.0)	3 (14.3)	15 (12.7)	34 (26.8)	114 (35.0)	**≤0.001¶**

Supplementary Table 3: continued

	**N1**	82 (13.9)	5 (23.8)	30 (25.4)	13 (10.2)	34 (10.4)	
	**N2-N2a**	58 (9.8)	2 (9.5)	19 (16.1)	10 (7.9)	27 (8.3)	
	**N2b**	184 (31.1)	7 (33.3)	42 (35.6)	45 (35.4)	90 (27.6)	
	**N2c**	79 (13.3)	4 (19.0)	8 (6.8)	21 (16.5)	46 (14.1)	
	**N3**	23 (3.9)	0 (0.0)	4 (3.4)	4 (3.1)	15 (4.6)	
	**unknown**	8	0	0	2	6	
**M-stage**	**M0**	539 (92.9)	16 (80.0)	111 (99.1)	105 (82.7)	313 (97.5)	**≤0.001**
	**M+**	41 (7.1)	4 (20.0)	1 (0.9)	22 (17.3)	8 (2.5)	
	**unknown**	20	1	6	2	11	
**Surgery**	**yes**	345	10	85	70	180	**≤0.001**
	**no**	250	10	33	57	150	
	**unknown**	5	1	0	2	2	
**Radio-therapy**	**yes**	447	13	102	101	231	**≤0.001**
	**no**	119	7	12	22	79	
	**Unknown**	34	1	4	6	22	
**Chemo-therapy**	**yes**	309	13	61	73	163	0,055
	**no**	265	7	52	51	154	
	**unknown**	26	1	5	5	15	

Patients without LDR were diagnosed between 2000 and before November 2014, patients with LDR were included until 2018. Percentage based on total cases (n=600) without missing valus. P-values (asymptotic, 2-sided) calculated by Pearson's chi square, (#) one-factorial ANOVA, (¶) Mantel-Haenszel test of trend or (§) Kruskal-Wallis test; p-values ≤0.05 in bold.
