# Supplementary material for: Mutation patterns in recurrent and/or metastatic oropharyngeal squamous cell carcinomas in relation to human papillomavirus status
Source: Cancer Med. 2021 Feb 1;10(4):1347–56. doi: 10.1002/cam4.3741 (PMC7926014; doi:10.1002/cam4.3741)
Supplement: Supplementary file 5 — Table S4 [file CAM4-10-1347-s005.pdf]

Supplementary Table 4: P-values of the mutation frequencies between HPV- OPSCC of patients with LDR and HPV- OPSCC of patients without LDR.

	**HPV- OPSCC of patients with LDR**	**HPV- OPSCC of patients without LDR**
TP53	0.540	
RB1	0.204	
STK11	0.431	
CDH1	0.274	
HRAS	0.191	
KRAS	0.336	
NRAS	0.193	
FAT1	**0.043**	
PIK3CA	0.558	
PIK3R1	0.100	
PTEN	0.130	
FANCA	0.334	
FBXW7	0.227	
CYLD	0.630	
BCL6	0.184	
TP63	0.201	
TAF1	0.183	
EP300	0.214	
DDX3X	0.321	
NOTCH1	0.202	
JAK1	0.521	
JAK2	0.130	
PDGFRA	0.405	

P-values were calculated by Mann-Whitney-U test for independent samples; p-values ≤0.05 in bold.
