# Supplementary material for: Mutation patterns in recurrent and/or metastatic oropharyngeal squamous cell carcinomas in relation to human papillomavirus status
Source: Cancer Med. 2021 Feb 1;10(4):1347–56. doi: 10.1002/cam4.3741 (PMC7926014; doi:10.1002/cam4.3741)
Supplement: Supplementary file 6 — Table S5 [file CAM4-10-1347-s006.pdf]

Supplementary Table 5: List of all detected mutations in each gene for all analyzed patients with a primary OPSCC.

HPV-status	LDR-status	Matching pair	Gene	Chr	Position	Mutation	rsID
negative	negative	08	HRAS	11	533496	NM_176795:c.407G>A, p.Ser136Asn	
negative	negative	02	HRAS	11	533530	NM_176795:c.373G>A, p.Val125Met	
negative	negative	08	HRAS	11	533538	NM_176795:c.365C>T, p.Ala122Val	rs779711606
positive	positive	02	HRAS	11	533539	NM_176795:c.364G>A, p.Ala122Thr	
positive	positive	01	HRAS	11	533548	NM_176795:c.355G>A, p.Asp119Asn	
positive	positive	11	HRAS	11	533550	NM_176795:c.353G>A, p.Cys118Tyr	
negative	negative	03	HRAS	11	533552	NM_176795:c.351G>T, p.Lys117Asn	
positive	positive	02	HRAS	11	533560	NM_176795:c.343G>A, p.Gly115Arg	rs917210997
negative	negative	08	HRAS	11	533570	NM_176795:c.333G>A, p.Met111Ile	
negative	negative	06	HRAS	11	533575	NM_176795:c.328C>T, p.Pro110Ser	
negative	negative	06	HRAS	11	533578	NM_176795:c.325G>A, p.Val109Met	rs1342261234
negative	positive	11	HRAS	11	533590	NM_176795:c.313G>A, p.Asp105Asn	rs1274939921
positive	positive	02	HRAS	11	533815	NM_176795:c.241G>A, p.Val81Met	
negative	positive	11	HRAS	11	533824	NM_176795:c.232T>C, p.Phe78Leu	
positive	positive	05	HRAS	11	533881	NM_176795:c.175G>A, p.Ala59Thr	rs727503093
negative	positive	11	HRAS	11	534226	NM_176795:c.97G>A, p.Asp33Asn	
negative	negative	06	HRAS	11	534235	NM_176795:c.88G>A, p.Asp30Asn	rs1226305644
positive	positive	01	HRAS	11	534273	NM_176795:c.50G>A, p.Ser17Asn	
positive	positive	06	HRAS	11	534286	NM_176795:c.37G>C, p.Gly13Arg	rs104894228
positive	positive	02	HRAS	11	534289	NM_176795:c.34G>A, p.Gly12Ser	rs104894229

Supplementary Table 5: continued

HPV-status	LDR-status	Matching pair	Gene	Chr	Position	Mutation	rsID
negative	positive	11	HRAS	11	534301	NM_176795:c.22G>A, p.Val8Met	
negative	positive	11	HRAS	11	534304	NM_176795:c.19G>A, p.Val7Met	
negative	positive	14	STK11	19	1220391	NM_000455:c.484G>A, p.Asp162Asn	
positive	positive	02	STK11	19	1220394	NM_000455:c.487G>A, p.Gly163Ser	
positive	positive	05	STK11	19	1220413	NM_000455:c.506G>A, p.Ser169Asn	
negative	negative	08	STK11	19	1220428	NM_000455:c.521A>G, p.His174Arg	
negative	negative	03	STK11	19	1220461	NM_000455:c.554C>T, p.Thr185Ile	rs540627331
negative	positive	10	STK11	19	1220470	NM_000455:c.563G>A, p.Gly188Asp	
negative	negative	02	STK11	19	1220487	NM_000455:c.580G>A, p.Asp194Asn	rs121913315
negative	negative	03	STK11	19	1220499	NM_000455:c.592G>A, p.Ala198Thr	
positive	positive	02	STK11	19	1220500	NM_000455:c.593C>T, p.Ala198Val	
negative	negative	06	STK11	19	1220502	NM_000455:c.595G>A, p.Glu199Lys	rs121913317
positive	positive	12	STK11	19	1221226	NM_000455:c.749C>T, p.Thr250Met	rs876661238
negative	negative	08	STK11	19	1221228	NM_000455:c.751G>A, p.Gly251Ser	rs748112446
negative	negative	06	STK11	19	1221229	NM_000455:c.752G>A, p.Gly251Asp	rs730881977
positive	positive	02	STK11	19	1221243	NM_000455:c.766G>A, p.Glu256Lys	
positive	positive	01	STK11	19	1221246	NM_000455:c.769G>A, p.Gly257Arg	
positive	positive	01	STK11	19	1221247	NM_000455:c.770G>A, p.Gly257Glu	rs1555738634
negative	negative	02	STK11	19	1221279	NM_000455:c.802G>A, p.Gly268Arg	
negative	negative	03	STK11	19	1221300	NM_000455:c.823C>T, p.Pro275Ser	
positive	positive	01	STK11	19	1221301	NM_000455:c.824C>T, p.Pro275Leu	rs1555738685

Supplementary Table 5: continued

HPV-status	LDR-status	Matching pair	Gene	Chr	Position	Mutation	rsID
negative	positive	11	STK11	19	1221303	NM_000455:c.826G>A, p.Gly276Ser	rs1461580063
positive	positive	02	STK11	19	1221310	NM_000455:c.833G>T, p.Cys278Phe	
positive	positive	01	STK11	19	1221313	NM_000455:c.836G>A, p.Gly279Asp	rs865941858
positive	positive	12	STK11	19	1221313	NM_000455:c.836G>A, p.Gly279Asp	rs865941858
negative	negative	08	JAK2	9	5064934	NM_004972:c.1108G>A, p.Asp370Asn	
negative	positive	10	JAK2	9	5064934	NM_004972:c.1108G>A, p.Asp370Asn	
positive	positive	01	JAK2	9	5064947	NM_004972:c.1121G>A, p.Arg374Lys	
negative	negative	02	JAK2	9	5064967	NM_004972:c.1141C>T, p.His381Tyr	rs774526479
negative	negative	05	JAK2	9	5064967	NM_004972:c.1141C>T, p.His381Tyr	rs774526479
negative	negative	08	JAK2	9	5064973	NM_004972:c.1147C>T, p.Leu383Phe	
positive	positive	12	JAK2	9	5064994	NM_004972:c.1168C>T, p.Pro390Ser	rs1406407961
negative	negative	08	JAK2	9	5065015	NM_004972:c.1189C>T, p.Gln397*	
negative	negative	08	JAK2	9	5066689	NM_004972:c.1226C>T, p.Ala409Val	
positive	positive	02	JAK2	9	5066695	NM_004972:c.1232G>A, p.Ser411Asn	
positive	positive	02	JAK2	9	5066713	NM_004972:c.1250G>A, p.Gly417Asp	rs778050966
negative	negative	03	JAK2	9	5066746	NM_004972:c.1283G>A, p.Ser428Asn	
negative	negative	02	JAK2	9	5066748	NM_004972:c.1285C>T, p.Pro429Ser	
positive	positive	01	JAK2	9	5066749	NM_004972:c.1286C>T, p.Pro429Leu	
negative	negative	02	JAK2	9	5066749	NM_004972:c.1286C>T, p.Pro429Leu	
positive	positive	01	JAK2	9	5066782	NM_004972:c.1319C>T, p.Ala440Val	
negative	positive	10	TP53	17	7572934	NM_000546:c.1175C>T, p.Ser392Leu	

Supplementary Table 5: continued

HPV-status	LDR-status	Matching pair	Gene	Chr	Position	Mutation	rsID
positive	positive	01	TP53	17	7572943	NM_000546:c.1166G>A, p.Gly389Glu	
positive	positive	05	TP53	17	7572943	NM_000546:c.1166G>A, p.Gly389Glu	
negative	negative	03	TP53	17	7572959	NM_000546:c.1150A>G, p.Met384Val	rs730882009
positive	positive	01	TP53	17	7572971	NM_000546:c.1138C>T, p.His380Tyr	
positive	positive	01	TP53	17	7572974	NM_000546:c.1135C>T, p.Arg379Cys	rs749061599
positive	positive	02	TP53	17	7572974	NM_000546:c.1135C>T, p.Arg379Cys	rs749061599
positive	positive	01	TP53	17	7572979	NM_000546:c.1130C>T, p.Thr377Ile	rs1597345549
negative	negative	06	TP53	17	7572999	NM_000546:c.61G>A, p.Val21Ile	rs1567540533
negative	negative	06	TP53	17	7573008	NM_000546:c.52C>T, p.Pro18Ser	
negative	positive	07	TP53	17	7573008	NM_000546:c.52C>T, p.Pro18Ser	
negative	negative	02	TP53	17	7573934	NM_000546:c.1093C>T, p.His365Tyr	rs267605075
negative	positive	10	TP53	17	7573934	NM_000546:c.1093C>T, p.His365Tyr	rs267605075
negative	negative	02	TP53	17	7573939	NM_000546:c.1088G>A, p.Arg363Lys	rs876660285
negative	negative	08	TP53	17	7573942	NM_000546:c.1085G>A, p.Ser362Asn	rs768803947
negative	negative	06	TP53	17	7573949	NM_000546:c.1078G>A, p.Gly360Arg	rs786203298
negative	negative	06	TP53	17	7573963	NM_000546:c.1064C>T, p.Ala355Val	rs1555524382
negative	positive	03	TP53	17	7573964	NM_000546:c.1063G>A, p.Ala355Thr	rs1157427821
negative	positive	10	TP53	17	7573973	NM_000546:c.1054G>A, p.Asp352Asn	
negative	positive	04	TP53	17	7574002	NM_000546:c.1024del, p.Arg342fs	
positive	positive	12	TP53	17	7574003	NM_000546:c.1024C>T, p.Arg342*	rs730882029
negative	negative	03	TP53	17	7574007	NM_000546:c.1020G>A, p.Met340Ile	

Supplementary Table 5: continued

HPV-status	LDR-status	Matching pair	Gene	Chr	Position	Mutation	rsID
negative	negative	08	TP53	17	7576544	NM_000546:c.1034C>T, p.Ser345Leu	rs758194998
negative	positive	02	TP53	17	7576546	NM_000546:c.1031dup, p.Asn344fs	
negative	negative	13	TP53	17	7576546	NM_000546:c.1031dup, p.Asn344fs	
negative	negative	08	TP53	17	7576582	NM_000546:c.996G>A, p.Met332Ile	
negative	negative	05	TP53	17	7576650	NM_000546:c.1001C>T, p.Thr334Ile	
negative	negative	02	TP53	17	7576870	NM_000546:c.976G>A, p.Glu326Lys	
negative	negative	03	TP53	17	7576897	NM_000546:c.949C>T, p.Gln317*	rs764735889
negative	negative	02	TP53	17	7576905	NM_000546:c.941C>T, p.Ser314Phe	rs751440465
negative	negative	05	TP53	17	7577021	NM_000546:c.917G>A, p.Arg306Gln	rs1048095040
positive	positive	02	TP53	17	7577033	NM_000546:c.905G>A, p.Gly302Glu	rs1060501202
negative	positive	08	TP53	17	7577046	NM_000546:c.892G>T, p.Glu298*	rs201744589
positive	positive	02	TP53	17	7577093	NM_000546:c.845G>A, p.Arg282Gln	rs730882008
negative	positive	03	TP53	17	7577104	NM_000546:c.820_833del, p.Val274fs	
negative	negative	08	TP53	17	7577105	NM_000546:c.833C>T, p.Pro278Leu	rs876659802
positive	positive	02	TP53	17	7577108	NM_000546:c.830G>A, p.Cys277Tyr	rs763098116
negative	positive	07	TP53	17	7577114	NM_000546:c.824G>T, p.Cys275Phe	rs863224451
negative	positive	11	TP53	17	7577114	NM_000546:c.824G>A, p.Cys275Tyr	rs863224451
positive	positive	02	TP53	17	7577120	NM_000546:c.818G>A, p.Arg273His	rs28934576
negative	negative	03	TP53	17	7577121	NM_000546:c.817C>T, p.Arg273Cys	rs121913343
negative	negative	03	TP53	17	7577139	NM_000546:c.799C>T, p.Arg267Trp	rs55832599
negative	positive	02	TP53	17	7577144	NM_000546:c.793_794insG, p.Leu265fs	

Supplementary Table 5: continued

HPV-status	LDR-status	Matching pair	Gene	Chr	Position	Mutation	rsID
negative	negative	13	TP53	17	7577144	NM_000546:c.793_794insG, p.Leu265fs	
negative	negative	06	TP53	17	7577499	NM_000546:c.782G>A, p.Ser261Asn	
negative	positive	14	TP53	17	7577499	NM_000546:c.782G>A, p.Ser261Asn	
negative	negative	06	TP53	17	7577506	NM_000546:c.775G>A, p.Asp259Asn	
negative	negative	06	TP53	17	7577518	NM_000546:c.763A>T, p.Ile255Phe	rs1057519995
negative	negative	08	TP53	17	7577527	NM_000546:c.754C>T, p.Leu252Phe	
negative	negative	01	TP53	17	7577534	NM_000546:c.747G>T, p.Arg249Ser	rs28934571
negative	negative	04	TP53	17	7577534	NM_000546:c.747G>T, p.Arg249Ser	rs28934571
negative	positive	10	TP53	17	7577538	NM_000546:c.743G>T, p.Arg248Leu	rs11540652
negative	positive	12	TP53	17	7577539	NM_000546:c.742C>T, p.Arg248Trp	rs121912651
positive	positive	02	TP53	17	7577548	NM_000546:c.733G>A, p.Gly245Ser	rs28934575
positive	negative	04	TP53	17	7577548	NM_000546:c.733G>T, p.Gly245Cys	rs28934575
positive	positive	06	TP53	17	7577559	NM_000546:c.722C>T, p.Ser241Phe	rs28934573
negative	negative	11	TP53	17	7577577	NM_000546:c.699_703del, p.His233fs	
positive	positive	02	TP53	17	7577584	NM_000546:c.697C>T, p.His233Tyr	
negative	positive	09	TP53	17	7578205	NM_000546:c.644G>T, p.Ser215Ile	rs587782177
negative	positive	06	TP53	17	7578235	NM_000546:c.614A>G, p.Tyr205Cys	rs1057520007
negative	positive	11	TP53	17	7578254	NM_000546:c.595G>A, p.Gly199Arg	
negative	negative	12	TP53	17	7578263	NM_000546:c.586C>T, p.Arg196*	rs397516435
negative	negative	05	TP53	17	7578266	NM_000546:c.583A>T, p.Ile195Phe	rs942158624
positive	positive	02	TP53	17	7578284	NM_000546:c.565G>A, p.Ala189Thr	

Supplementary Table 5: continued

HPV-status	LDR-status	Matching pair	Gene	Chr	Position	Mutation	rsID
negative	positive	10	TP53	17	7578374	NM_000546:c.556G>A, p.Asp186Asn	rs1060501206
positive	positive	01	TP53	17	7578380	NM_000546:c.550G>A, p.Asp184Asn	rs72661117
positive	positive	06	TP53	17	7578389	NM_000546:c.541C>T, p.Arg181Cys	rs587782596
negative	positive	02	TP53	17	7578393	NM_000546:c.536_537insC, p.Glu180fs	
negative	negative	13	TP53	17	7578393	NM_000546:c.536_537insC, p.Glu180fs	
positive	positive	06	TP53	17	7578400	NM_000546:c.530C>T, p.Pro177Leu	rs751477326
negative	negative	07	TP53	17	7578406	NM_000546:c.524G>A, p.Arg175His	rs28934578
positive	positive	06	TP53	17	7578407	NM_000546:c.523C>T, p.Arg175Cys	rs138729528
negative	positive	10	TP53	17	7578409	NM_000546:c.521G>A, p.Arg174Lys	rs1064796681
positive	positive	06	TP53	17	7578419	NM_000546:c.511G>A, p.Glu171Lys	rs587781845
negative	positive	13	TP53	17	7578419	NM_000546:c.511G>T, p.Glu171*	rs587781845
negative	positive	10	TP53	17	7578421	NM_000546:c.509C>T, p.Thr170Met	rs779000871
negative	negative	07	TP53	17	7578423	NM_000546:c.507G>A, p.Met169Ile	
negative	negative	03	TP53	17	7578445	NM_000546:c.485T>A, p.Ile162Asn	
negative	negative	02	TP53	17	7578448	NM_000546:c.482C>T, p.Ala161Val	
negative	negative	06	TP53	17	7578450	NM_000546:c.480G>A, p.Met160Ile	rs772354334
negative	negative	06	TP53	17	7578458	NM_000546:c.472C>T, p.Arg158Cys	rs587780068
negative	positive	09	TP53	17	7578461	NM_000546:c.469G>T, p.Val157Phe	rs121912654
positive	positive	02	TP53	17	7578472	NM_000546:c.458C>T, p.Pro153Leu	rs1597370799
positive	positive	01	TP53	17	7578473	NM_000546:c.457C>T, p.Pro153Ser	rs1064795860
negative	negative	14	TP53	17	7578478	NM_000546:c.452C>G, p.Pro151Arg	rs1057520000

Supplementary Table 5: continued

HPV-status	LDR-status	Matching pair	Gene	Chr	Position	Mutation	rsID
negative	negative	06	TP53	17	7578479	NM_000546:c.451C>T, p.Pro151Ser	rs28934874
negative	negative	02	TP53	17	7578481	NM_000546:c.449C>T, p.Thr150Ile	
positive	positive	01	TP53	17	7578488	NM_000546:c.442G>A, p.Asp148Asn	rs1131691007
negative	negative	08	TP53	17	7578491	NM_000546:c.439G>A, p.Val147Ile	rs1555526226
negative	negative	02	TP53	17	7578500	NM_000546:c.430C>T, p.Gln144*	rs757274881
negative	negative	05	TP53	17	7578509	NM_000546:c.420del, p.Cys141fs	
negative	negative	02	TP53	17	7578524	NM_000546:c.406C>T, p.Gln136*	
negative	positive	03	TP53	17	7578524	NM_000546:c.406C>T, p.Gln136*	
negative	positive	10	TP53	17	7578526	NM_000546:c.404G>A, p.Cys135Tyr	rs587781991
positive	positive	01	TP53	17	7578544	NM_000546:c.386C>T, p.Ala129Val	rs137852792
negative	negative	08	TP53	17	7578544	NM_000546:c.386C>T, p.Ala129Val	rs137852792
negative	negative	02	TP53	17	7579325	NM_000546:c.362C>T, p.Ser121Phe	
negative	negative	03	TP53	17	7579340	NM_000546:c.347C>T, p.Ser116Phe	
negative	negative	06	TP53	17	7579340	NM_000546:c.347C>T, p.Ser116Phe	
negative	negative	06	TP53	17	7579352	NM_000546:c.335G>A, p.Gly112Asp	
negative	negative	08	TP53	17	7579359	NM_000546:c.328C>T, p.Arg110Cys	rs587781371
positive	positive	01	TP53	17	7579377	NM_000546:c.310C>T, p.Gln104*	rs1567555934
negative	negative	06	TP53	17	7579395	NM_000546:c.292C>T, p.Pro98Ser	rs1597374015
negative	negative	06	TP53	17	7579412	NM_000546:c.275C>T, p.Pro92Leu	rs1210700121
negative	positive	03	TP53	17	7579413	NM_000546:c.274C>T, p.Pro92Ser	
negative	negative	08	TP53	17	7579418	NM_000546:c.269C>T, p.Ser90Phe	rs1555526625

Supplementary Table 5: continued

HPV-status	LDR-status	Matching pair	Gene	Chr	Position	Mutation	rsID
negative	negative	08	TP53	17	7579421	NM_000546:c.266C>T, p.Pro89Leu	rs730881994
negative	positive	14	TP53	17	7579423	NM_000546:c.234_263del, p.Ala79_Ala88del	
negative	negative	02	TP53	17	7579427	NM_000546:c.260C>T, p.Pro87Leu	
negative	negative	02	TP53	17	7579428	NM_000546:c.259C>T, p.Pro87Ser	
negative	negative	06	TP53	17	7579428	NM_000546:c.259C>T, p.Pro87Ser	
negative	negative	08	TP53	17	7579463	NM_000546:c.224C>T, p.Pro75Leu	
negative	positive	11	TP53	17	7579515	NM_000546:c.172C>T, p.Pro58Ser	
negative	negative	03	TP53	17	7579528	NM_000546:c.159G>A, p.Trp53*	rs1064794618
negative	positive	11	TP53	17	7579529	NM_000546:c.158G>A, p.Trp53*	rs876658483
positive	negative	01	TP53	17	7579532	NM_000546:c.154dup, p.Gln52fs	
negative	negative	03	TP53	17	7579590	NM_000546:c.97T>G, p.Ser33Ala	
negative	negative	08	TP53	17	7579869	NM_000546:c.44G>A, p.Ser15Asn	
negative	negative	03	TP53	17	7579875	NM_000546:c.38C>T, p.Pro13Leu	rs878854070
negative	negative	02	TP53	17	7579876	NM_000546:c.37C>T, p.Pro13Ser	rs1060501208
negative	positive	11	TP53	17	7579876	NM_000546:c.37C>T, p.Pro13Ser	rs1060501208
negative	negative	02	TP53	17	7579882	NM_000546:c.31G>A, p.Glu11Lys	rs201382018
negative	negative	02	KRAS	12	25398210	NM_033360:c.109G>A, p.Glu37Lys	
negative	negative	07	KRAS	12	25398222	NM_033360:c.97G>A, p.Asp33Asn	
negative	negative	02	KRAS	12	25398234	NM_033360:c.85G>A, p.Val29Met	
negative	positive	10	KRAS	12	25398255	NM_033360:c.64C>T, p.Gln22*	
negative	positive	02	KRAS	12	25398267	NM_033360:c.52G>A, p.Ala18Thr	

Supplementary Table 5: continued

HPV-status	LDR-status	Matching pair	Gene	Chr	Position	Mutation	rsID
negative	negative	02	KRAS	12	25398267	NM_033360:c.52G>A, p.Ala18Thr	
positive	negative	03	KRAS	12	25398269	NM_033360:c.50G>A, p.Ser17Asn	
negative	negative	05	KRAS	12	25398269	NM_033360:c.50G>A, p.Ser17Asn	
negative	negative	05	KRAS	12	25398276	NM_033360:c.43G>A, p.Gly15Ser	
negative	negative	03	KRAS	12	25398281	NM_033360:c.38G>A, p.Gly13Asp	rs112445441
negative	negative	05	KRAS	12	25398281	NM_033360:c.38G>A, p.Gly13Asp	rs112445441
negative	positive	10	KRAS	12	25398287	NM_033360:c.32C>T, p.Ala11Val	
negative	negative	02	KRAS	12	25398297	NM_033360:c.22G>A, p.Val8Ile	
negative	negative	08	DDX3X	X	41193537	NM_001193416:c.32G>A, p.Gly11Glu	
negative	negative	08	DDX3X	X	41200802	NM_001193416:c.217G>A, p.Gly73Arg	
negative	positive	10	DDX3X	X	41200803	NM_001193416:c.218G>A, p.Gly73Glu	
negative	positive	02	DDX3X	X	41200808	NM_001193416:c.223C>T, p.Arg75Cys	rs1289923555
negative	negative	03	DDX3X	X	41200812	NM_001193416:c.227G>A, p.Ser76Asn	
negative	positive	12	DDX3X	X	41200814	NM_001193416:c.229G>A, p.Asp77Asn	
negative	negative	03	DDX3X	X	41200818	NM_001193416:c.233C>T, p.Ser78Leu	
positive	positive	06	DDX3X	X	41200821	NM_001193416:c.236G>A, p.Arg79Lys	rs1064795656
positive	negative	04	DDX3X	X	41200823	NM_001193416:c.238G>A, p.Gly80Arg	
negative	positive	02	DDX3X	X	41200824	NM_001193416:c.239G>A, p.Gly80Glu	
negative	negative	06	DDX3X	X	41200824	NM_001193416:c.239G>A, p.Gly80Glu	
negative	positive	02	DDX3X	X	41200833	NM_001193416:c.248G>A, p.Ser83Asn	
negative	negative	05	DDX3X	X	41200844	NM_001193416:c.259G>A, p.Asp87Asn	

Supplementary Table 5: continued

HPV-status	LDR-status	Matching pair	Gene	Chr	Position	Mutation	rsID
positive	positive	05	DDX3X	X	41200850	NM_001193416:c.265G>A, p.Gly89Arg	
negative	positive	02	DDX3X	X	41200854	NM_001193416:c.269G>A, p.Ser90Asn	
negative	positive	10	DDX3X	X	41200857	NM_001193416:c.272G>A, p.Gly91Glu	
negative	negative	05	DDX3X	X	41200860	NM_001193416:c.275C>T, p.Ser92Leu	
positive	positive	05	DDX3X	X	41200863	NM_001193416:c.278G>A, p.Arg93Lys	
negative	negative	06	DDX3X	X	41201991	NM_001193416:c.445G>A, p.Glu149Lys	
negative	negative	06	DDX3X	X	41201991	NM_001193416:c.445G>A, p.Glu149Lys	
negative	negative	07	DDX3X	X	41201991	NM_001193416:c.445G>A, p.Glu149Lys	
positive	positive	01	DDX3X	X	41202003	NM_001193416:c.457G>A, p.Gly153Arg	
negative	negative	07	DDX3X	X	41202003	NM_001193416:c.457G>A, p.Gly153Arg	
negative	negative	05	DDX3X	X	41202013	NM_001193416:c.467C>T, p.Thr156Ile	
negative	negative	06	DDX3X	X	41202013	NM_001193416:c.467C>T, p.Thr156Ile	
negative	negative	07	DDX3X	X	41202013	NM_001193416:c.467C>T, p.Thr156Ile	
negative	positive	10	DDX3X	X	41202015	NM_001193416:c.469G>A, p.Gly157Arg	
negative	negative	06	DDX3X	X	41202027	NM_001193416:c.481G>A, p.Glu161Lys	
negative	negative	07	DDX3X	X	41202027	NM_001193416:c.481G>A, p.Glu161Lys	
positive	positive	06	DDX3X	X	41202036	NM_001193416:c.490G>A, p.Asp164Asn	
negative	negative	03	DDX3X	X	41202048	NM_001193416:c.502G>A, p.Val168Ile	
negative	negative	05	DDX3X	X	41202048	NM_001193416:c.502G>A, p.Val168Ile	
negative	positive	02	DDX3X	X	41202988	NM_001193416:c.681del, p.Ser228fs	
negative	negative	13	DDX3X	X	41202988	NM_001193416:c.681del, p.Ser228fs	

Supplementary Table 5: continued

HPV-status	LDR-status	Matching pair	Gene	Chr	Position	Mutation	rsID
positive	positive	05	DDX3X	X	41202995	NM_001193416:c.685G>A, p.Gly229Arg	
negative	positive	02	DDX3X	X	41203004	NM_001193416:c.694_695insA, p.Ala232fs	
negative	negative	13	DDX3X	X	41203004	NM_001193416:c.694_695insA, p.Ala232fs	
negative	negative	08	DDX3X	X	41203019	NM_001193416:c.709C>T, p.Pro237Ser	
positive	positive	06	DDX3X	X	41203028	NM_001193416:c.718A>G, p.Ser240Gly	
negative	negative	03	DDX3X	X	41203029	NM_001193416:c.719G>A, p.Ser240Asn	
negative	negative	02	DDX3X	X	41203041	NM_001193416:c.731C>T, p.Ser244Leu	rs1275844963
negative	negative	08	DDX3X	X	41203041	NM_001193416:c.731C>T, p.Ser244Leu	rs1275844963
negative	positive	11	DDX3X	X	41206159	NM_001193416:c.1663G>A, p.Asp555Asn	
negative	negative	02	DDX3X	X	41206177	NM_001193416:c.1681G>A, p.Val561Ile	
negative	negative	07	DDX3X	X	41206177	NM_001193416:c.1681G>A, p.Val561Ile	
negative	negative	07	DDX3X	X	41206189	NM_001193416:c.1693C>T, p.Gln565*	
positive	positive	12	DDX3X	X	41206192	NM_001193416:c.1696G>A, p.Glu566Lys	
negative	negative	02	DDX3X	X	41206240	NM_001193416:c.1744G>A, p.Gly582Ser	
negative	negative	08	EP300	22	41545049	NM_001429:c.2249G>A, p.Gly750Asp	rs1433543827
negative	negative	08	EP300	22	41545054	NM_001429:c.2254G>A, p.Gly752Arg	rs1338244902
negative	negative	08	EP300	22	41545055	NM_001429:c.2255G>A, p.Gly752Glu	
positive	positive	01	EP300	22	41545057	NM_001429:c.2257C>T, p.Pro753Ser	
negative	negative	08	EP300	22	41545060	NM_001429:c.2260C>T, p.Arg754Cys	
negative	positive	02	EP300	22	41545064	NM_001429:c.2265dup, p.Gln756fs	
negative	negative	13	EP300	22	41545064	NM_001429:c.2265dup, p.Gln756fs	

Supplementary Table 5: continued

HPV-status	LDR-status	Matching pair	Gene	Chr	Position	Mutation	rsID
negative	negative	08	EP300	22	41545066	NM_001429:c.2266C>T, p.Gln756*	
negative	negative	08	EP300	22	41545087	NM_001429:c.2287C>T, p.Gln763*	
negative	negative	08	EP300	22	41545093	NM_001429:c.2293C>T, p.Leu765Phe	
positive	negative	03	EP300	22	41545097	NM_001429:c.2297C>T, p.Pro766Leu	rs760686401
positive	positive	01	EP300	22	41545111	NM_001429:c.2311C>T, p.Pro771Ser	
negative	positive	10	EP300	22	41545115	NM_001429:c.2315C>T, p.Ser772Leu	rs776869457
negative	negative	02	EP300	22	41553350	NM_001429:c.3439G>A, p.Glu1147Lys	
negative	positive	10	EP300	22	41553353	NM_001429:c.3442G>A, p.Val1148Ile	rs750777340
negative	negative	03	EP300	22	41553359	NM_001429:c.3448G>A, p.Glu1150Lys	
positive	negative	03	EP300	22	41553362	NM_001429:c.3451C>T, p.Gln1151*	
negative	positive	10	EP300	22	41553377	NM_001429:c.3466G>A, p.Val1156Met	
negative	positive	10	EP300	22	41553399	NM_001429:c.3488G>A, p.Cys1163Tyr	
negative	positive	10	EP300	22	41553402	NM_001429:c.3491G>A, p.Cys1164Tyr	
positive	negative	03	EP300	22	41565521	NM_001429:c.4187C>T, p.Ser1396Phe	
negative	negative	08	EP300	22	41565521	NM_001429:c.4187C>T, p.Ser1396Phe	
negative	positive	10	EP300	22	41565526	NM_001429:c.4192C>T, p.Leu1398Phe	
negative	negative	02	EP300	22	41565529	NM_001429:c.4195G>T, p.Asp1399Tyr	rs1057519889
positive	positive	02	EP300	22	41565538	NM_001429:c.4204C>T, p.His1402Tyr	rs1048138769
positive	negative	03	EP300	22	41565538	NM_001429:c.4204C>T, p.His1402Tyr	rs1048138769
negative	negative	06	EP300	22	41565620	NM_001429:c.4286G>A, p.Gly1429Asp	
negative	negative	08	EP300	22	41566433	NM_001429:c.4310C>T, p.Ala1437Val	rs769217956

Supplementary Table 5: continued

HPV-status	LDR-status	Matching pair	Gene	Chr	Position	Mutation	rsID
negative	negative	08	EP300	22	41566439	NM_001429:c.4316C>T, p.Pro1439Leu	
positive	positive	02	EP300	22	41566468	NM_001429:c.4345C>T, p.His1449Tyr	
negative	negative	08	EP300	22	41566472	NM_001429:c.4349G>A, p.Cys1450Tyr	
positive	positive	12	EP300	22	41566501	NM_001429:c.4378C>T, p.Pro1460Ser	
positive	positive	12	EP300	22	41566516	NM_001429:c.4393G>A, p.Glu1465Lys	
negative	negative	08	RB1	13	48936999	NM_000321:c.767G>A, p.Gly256Asp	rs770857159
negative	negative	02	RB1	13	48937047	NM_000321:c.815G>A, p.Arg272Lys	
negative	negative	02	RB1	13	48937058	NM_000321:c.826G>A, p.Val276Ile	
negative	negative	08	RB1	13	48937080	NM_000321:c.848G>A, p.Cys283Tyr	rs1273219762
negative	negative	03	RB1	13	49027129	NM_000321:c.1696G>A, p.Asp566Asn	
negative	positive	10	RB1	13	49027135	NM_000321:c.1702C>T, p.Pro568Ser	rs754634473
negative	negative	03	RB1	13	49027177	NM_000321:c.1744C>T, p.Pro582Ser	rs968643096
negative	negative	07	RB1	13	49027178	NM_000321:c.1745C>T, p.Pro582Leu	
negative	positive	02	RB1	13	49027186	NM_000321:c.1753C>T, p.His585Tyr	rs1401332173
positive	positive	01	RB1	13	49027199	NM_000321:c.1766C>T, p.Ala589Val	rs779045727
negative	negative	07	RB1	13	49027205	NM_000321:c.1772C>T, p.Pro591Leu	rs1221157259
negative	positive	10	RB1	13	49027207	NM_000321:c.1774C>T, p.Leu592Phe	
negative	negative	07	RB1	13	49027217	NM_000321:c.1784C>T, p.Pro595Leu	rs772068738
negative	negative	05	RB1	13	49027219	NM_000321:c.1786C>T, p.Leu596Phe	
negative	negative	07	RB1	13	49033883	NM_000321:c.2020C>T, p.Pro674Ser	
positive	positive	01	RB1	13	49033892	NM_000321:c.2029G>A, p.Glu677Lys	

Supplementary Table 5: continued

HPV-status	LDR-status	Matching pair	Gene	Chr	Position	Mutation	rsID
negative	positive	10	RB1	13	49033895	NM_000321:c.2032C>T, p.His678Tyr	
negative	negative	02	RB1	13	49033905	NM_000321:c.2042G>A, p.Trp681*	
negative	negative	03	RB1	13	49033905	NM_000321:c.2042G>A, p.Trp681*	
negative	positive	10	RB1	13	49033940	NM_000321:c.2077G>A, p.Glu693Lys	
positive	positive	01	RB1	13	49033952	NM_000321:c.2089G>A, p.Asp697Asn	
negative	positive	10	CYLD	16	50811804	NM_015247:c.1090G>A, p.Asp364Asn	
negative	positive	10	CYLD	16	50811808	NM_015247:c.1094C>T, p.Ser365Leu	
positive	positive	02	CYLD	16	50811813	NM_015247:c.1099C>T, p.Pro367Ser	
negative	negative	03	CYLD	16	50811839	NM_015247:c.1125G>A, p.Trp375*	
negative	negative	05	CYLD	16	50811849	NM_015247:c.1135G>A, p.Glu379Lys	
negative	positive	11	CYLD	16	50818283	NM_015247:c.1870C>T, p.Pro624Ser	
negative	positive	10	CYLD	16	50821699	NM_015247:c.2044C>T, p.Pro682Ser	
negative	negative	07	PDGFRA	4	55141026	NM_006206:c.1672C>T, p.Arg558Cys	rs765680542
negative	negative	02	PDGFRA	4	55141031	NM_006206:c.1677G>A, p.Trp559*	
negative	positive	03	PDGFRA	4	55141031	NM_006206:c.1677G>A, p.Trp559*	
positive	positive	02	PDGFRA	4	55141033	NM_006206:c.1679G>A, p.Arg560Lys	
negative	positive	10	PDGFRA	4	55141053	NM_006206:c.1699C>T, p.Pro567Ser	
negative	positive	03	PDGFRA	4	55141060	NM_006206:c.1706G>A, p.Gly569Glu	
negative	negative	07	PDGFRA	4	55141083	NM_006206:c.1729C>T, p.Pro577Ser	rs1057519811
negative	negative	02	PDGFRA	4	55151567	NM_006206:c.1633G>A, p.Asp545Asn	
positive	positive	02	PDGFRA	4	55151576	NM_006206:c.2362G>A, p.Glu788Lys	

Supplementary Table 5: continued

HPV-status	LDR-status	Matching pair	Gene	Chr	Position	Mutation	rsID
negative	negative	02	PDGFRA	4	55151576	NM_006206:c.2362G>A, p.Glu788Lys	
negative	negative	02	PDGFRA	4	55151586	NM_006206:c.1652C>T, p.Thr551Ile	
negative	positive	10	PDGFRA	4	55151586	NM_006206:c.1652C>T, p.Thr551Ile	
negative	negative	02	PDGFRA	4	55151610	NM_006206:c.1676C>T, p.Thr559Ile	rs772948645
negative	negative	06	PDGFRA	4	55151624	NM_006206:c.1690C>T, p.Arg564*	rs1242960071
positive	positive	01	PDGFRA	4	55151628	NM_006206:c.2414G>A, p.Gly805Glu	
negative	negative	03	PDGFRA	4	55151632	NM_006206:c.2418G>A, p.Met806Ile	rs867690523
positive	negative	04	PDGFRA	4	55151632	NM_006206:c.2418G>A, p.Met806Ile	
negative	negative	06	JAK1	1	65330487	NM_002227:c.1159C>T, p.Gln387*	
negative	negative	02	JAK1	1	65330520	NM_002227:c.1126G>A, p.Val376Ile	
negative	positive	02	JAK1	1	65330528	NM_002227:c.1118C>T, p.Thr373Ile	
negative	negative	02	JAK1	1	65330562	NM_002227:c.1084G>A, p.Glu362Lys	
positive	positive	02	JAK1	1	65330565	NM_002227:c.1081G>A, p.Glu361Lys	
negative	negative	02	PIK3R1	5	67576358	NM_181523:c.637G>A, p.Val213Ile	rs150170197
negative	negative	05	PIK3R1	5	67576368	NM_181523:c.647C>T, p.Ser216Phe	
negative	negative	07	PIK3R1	5	67576407	NM_181523:c.686C>T, p.Ser229Leu	rs201598843
positive	positive	05	PIK3R1	5	67588092	NM_181523:c.922C>T, p.Pro308Ser	rs1561296591
negative	positive	10	PIK3R1	5	67588092	NM_181523:c.922C>T, p.Pro308Ser	rs1561296591
negative	positive	10	PIK3R1	5	67588096	NM_181523:c.926C>T, p.Pro309Leu	
positive	positive	06	PIK3R1	5	67588102	NM_181523:c.932C>T, p.Pro311Leu	
negative	negative	02	PIK3R1	5	67588104	NM_181523:c.934C>T, p.Pro312Ser	rs1293887048

Supplementary Table 5: continued

HPV-status	LDR-status	Matching pair	Gene	Chr	Position	Mutation	rsID
positive	positive	06	PIK3R1	5	67588104	NM_181523:c.934C>T, p.Pro312Ser	rs1293887048
negative	negative	07	PIK3R1	5	67588111	NM_181523:c.941C>T, p.Pro314Leu	
negative	negative	02	PIK3R1	5	67588119	NM_181523:c.949G>A, p.Val317Ile	rs766076215
positive	positive	01	PIK3R1	5	67588131	NM_181523:c.961G>A, p.Gly321Ser	rs142439210
positive	positive	01	PIK3R1	5	67588132	NM_181523:c.962G>A, p.Gly321Asp	
negative	negative	06	PIK3R1	5	67588132	NM_181523:c.962G>A, p.Gly321Asp	
negative	negative	07	PIK3R1	5	67588132	NM_181523:c.962G>A, p.Gly321Asp	
positive	positive	01	PIK3R1	5	67588150	NM_181523:c.980C>T, p.Ser327Phe	
negative	positive	10	PIK3R1	5	67588158	NM_181523:c.988G>A, p.Asp330Asn	
negative	negative	07	PIK3R1	5	67588164	NM_181523:c.994G>A, p.Glu332Lys	
positive	positive	01	PIK3R1	5	67588168	NM_181523:c.998G>A, p.Trp333*	
negative	negative	08	PIK3R1	5	67588168	NM_181523:c.998G>A, p.Trp333*	
positive	positive	01	PIK3R1	5	67588169	NM_181523:c.999G>A, p.Trp333*	
negative	negative	08	PIK3R1	5	67588175	NM_181523:c.1005G>A, p.Trp335*	
negative	negative	07	PIK3R1	5	67588179	NM_181523:c.1009G>A, p.Asp337Asn	
negative	negative	02	PIK3R1	5	67588189	NM_181523:c.1019G>A, p.Arg340Lys	
positive	positive	09	PIK3R1	5	67589602	NM_181523:c.1368dup, p.Gln457fs	
negative	negative	02	PIK3R1	5	67591077	NM_181523:c.1670G>A, p.Arg557Gln	
negative	positive	10	PIK3R1	5	67591077	NM_181523:c.1670G>A, p.Arg557Gln	
negative	negative	02	PIK3R1	5	67591085	NM_181523:c.1678G>A, p.Asp560Asn	
negative	negative	08	PIK3R1	5	67591085	NM_181523:c.1678G>A, p.Asp560Asn	

Supplementary Table 5: continued

HPV-status	LDR-status	Matching pair	Gene	Chr	Position	Mutation	rsID
negative	positive	02	PIK3R1	5	67591101	NM_181523:c.1694_1695insA, p.Ser565fs	
negative	negative	13	PIK3R1	5	67591101	NM_181523:c.1694_1695insA, p.Ser565fs	
negative	negative	07	PIK3R1	5	67591115	NM_181523:c.1708C>T, p.Leu570Phe	rs1561299896
positive	positive	01	PIK3R1	5	67591128	NM_181523:c.1721G>A, p.Arg574Lys	
negative	positive	10	CDH1	16	68846084	NM_004360:c.1055G>A, p.Gly352Asp	rs1555515728
negative	negative	08	CDH1	16	68846090	NM_004360:c.1061G>A, p.Gly354Glu	
negative	negative	03	CDH1	16	68846099	NM_004360:c.1070C>T, p.Thr357Ile	rs1260033180
negative	negative	03	CDH1	16	68846111	NM_004360:c.1082C>T, p.Ala361Val	
positive	positive	01	CDH1	16	68846120	NM_004360:c.1091C>T, p.Thr364Ile	rs876658376
negative	negative	03	CDH1	16	68846122	NM_004360:c.1093G>A, p.Val365Ile	rs1555515742
negative	negative	08	CDH1	16	68846126	NM_004360:c.1097C>T, p.Thr366Ile	
negative	negative	03	CDH1	16	68846144	NM_004360:c.1115C>T, p.Pro372Leu	rs1555515760
negative	negative	07	CDH1	16	68846146	NM_004360:c.1117C>T, p.Pro373Ser	
negative	positive	10	CDH1	16	68846147	NM_004360:c.1118C>T, p.Pro373Leu	rs587782359
positive	positive	01	CDH1	16	68846158	NM_004360:c.1129C>T, p.Pro377Ser	
negative	negative	08	CDH1	16	68846165	NM_004360c.1136C>T, p.Thr379Met	rs587782856
negative	negative	03	CDH1	16	68847234	NM_004360:c.1156G>A, p.Glu386Lys	
negative	negative	03	CDH1	16	68847259	NM_004360:c.1181C>T, p.Thr394Ile	rs1332444039
negative	negative	02	TAF1	X	70612744	NM_004606:c.2948C>T, p.Pro983Leu	
negative	negative	05	TAF1	X	70612746	NM_004606:c.2950G>A, p.Val984Met	
negative	negative	02	TAF1	X	70612758	NM_004606:c.2962G>A, p.Val988Met	

Supplementary Table 5: continued

HPV-status	LDR-status	Matching pair	Gene	Chr	Position	Mutation	rsID
negative	positive	10	TAF1	X	70612758	NM_004606:c.2962G>A, p.Val988Met	
positive	negative	04	TAF1	X	70612762	NM_004606:c.2966C>T, p.Thr989Ile	
positive	positive	02	TAF1	X	70612764	NM_004606:c.2968G>A, p.Gly990Arg	
negative	negative	12	TAF1	X	70612764	NM_004606:c.2968G>A, p.Gly990Arg	
negative	positive	03	TAF1	X	70612765	NM_004606:c.2969G>A, p.Gly990Glu	
positive	negative	04	TAF1	X	70612770	NM_004606:c.2974G>A, p.Asp992Asn	
negative	negative	08	TAF1	X	70612776	NM_004606:c.2980G>A, p.Asp994Asn	
positive	positive	01	TAF1	X	70612803	NM_004606:c.3007G>A, p.Ala1003Thr	
positive	positive	06	TAF1	X	70612818	NM_004606:c.3022C>T, p.Arg1008Cys	
negative	negative	02	TAF1	X	70612819	NM_004606:c.3023G>A, p.Arg1008His	
negative	negative	08	TAF1	X	70612827	NM_004606:c.3031G>A, p.Gly1011Ser	
positive	negative	04	TAF1	X	70612833	NM_004606:c.3037C>T, p.Pro1013Ser	
negative	positive	03	TAF1	X	70612836	NM_004606:c.3040G>A, p.Glu1014Lys	
negative	negative	05	TAF1	X	70612839	NM_004606:c.3043G>A, p.Glu1015Lys	
positive	positive	12	TAF1	X	70683756	NM_004606:c.5479G>A, p.Glu1827Lys	
positive	negative	10	TAF1	X	70683759	NM_004606:c.5482G>A, p.Glu1828Lys	
negative	negative	03	TAF1	X	70683762	NM_004606:c.5587G>A, p.Glu1863Lys	
positive	positive	01	TAF1	X	70683768	NM_004606:c.5491G>A, p.Asp1831Asn	
negative	negative	02	TAF1	X	70683771	NM_004606:c.5596G>A, p.Glu1866Lys	
positive	positive	01	TAF1	X	70683774	NM_004606:c.5497G>A, p.Glu1833Lys	
negative	negative	08	TAF1	X	70683774	NM_004606:c.5599G>A, p.Glu1867Lys	

Supplementary Table 5: continued

HPV-status	LDR-status	Matching pair	Gene	Chr	Position	Mutation	rsID
negative	positive	03	TAF1	X	70683777	NM_004606:c.5602G>A, p.Glu1868Lys	
positive	positive	02	TAF1	X	70683811	NM_004606:c.5534G>A, p.Ser1845Asn	rs1473260520
positive	positive	01	TAF1	X	70683828	NM_004606:c.5551G>A, p.Glu1851Lys	
negative	negative	08	TAF1	X	70683828	NM_004606:c.5551G>A, p.Glu1851Lys	
positive	positive	12	TAF1	X	70683831	NM_004606:c.5554G>A, p.Asp1852Asn	
negative	negative	08	TAF1	X	70683834	NM_004606:c.5659G>A, p.Glu1887Lys	rs1406838285
positive	positive	01	PTEN	10	89692790	NM_000314:c.274G>A, p.Asp92Asn	
positive	positive	01	PTEN	10	89692793	NM_000314:c.277C>T, p.His93Tyr	rs786204927
positive	positive	01	PTEN	10	89692799	NM_000314 c.283C>T, p.Pro95Ser	
positive	positive	02	PTEN	10	89692814	NM_000314:c.298C>T, p.Leu100Phe	
negative	negative	03	PTEN	10	89692823	NM_000314:c.307C>T, p.Pro103Ser	
positive	positive	01	PTEN	10	89692844	NM_000314:c.328C>T, p.Gln110*	rs1114167629
negative	negative	02	PTEN	10	89692848	NM_000314:c.328C>T, p.Trp111*	
negative	negative	05	PTEN	10	89692849	NM_000314:c.333G>A, p.Trp111*	rs1554898097
negative	positive	11	PTEN	10	89692868	NM_000314:c.352C>T, p.His118Tyr	rs1564830084
negative	negative	03	PTEN	10	89692887	NM_000314:c.371G>A, p.Cys124Tyr	rs876660535
negative	negative	08	PTEN	10	89692893	NM_000314:c.377C>T, p.Ala126Val	rs1114167656
negative	negative	02	PTEN	10	89692895	NM_000314:c.379G>A, p.Gly127Arg	rs587781255
negative	negative	03	PTEN	10	89717718	NM_000314:c.743C>T, p.Pro248Leu	
negative	negative	03	PTEN	10	89717750	NM_000314:c.775C>T, p.His259Tyr	
negative	negative	03	FANCA	16	89849269	NM_000135:c.1624G>A, p.Glu542Lys	

Supplementary Table 5: continued

HPV-status	LDR-status	Matching pair	Gene	Chr	Position	Mutation	rsID
negative	negative	06	FANCA	16	89849271	NM_000135:c.1622C>T, p.Thr541Ile	
negative	positive	10	FANCA	16	89849305	NM_000135:c.1588C>T, p.Leu530Phe	
negative	negative	06	FANCA	16	89849309	NM_000135:c.1584G>A, p.Met528Ile	
positive	positive	12	FANCA	16	89849322	NM_000135:c.1571C>T, p.Ser524Phe	
negative	negative	13	FANCA	16	89849322	NM_000135:c.1570del, p.Ser524fs	
negative	positive	06	FANCA	16	89858410	NM_000135:c.1150G>T, p.Val384Phe	rs751071791
positive	positive	01	FANCA	16	89877165	NM_000135:c.472C>T, p.His158Tyr	
negative	negative	08	FANCA	16	89877167	NM_000135:c.470C>T, p.Ala157Val	
positive	positive	01	FANCA	16	89877197	NM_000135:c.440C>T, p.Ser147Phe	rs138744489
negative	negative	03	NRAS	1	115256463	NM_002524:c.248C>T, p.Ala83Val	
positive	positive	01	NRAS	1	115256470	NM_002524:c.241G>A, p.Val81Ile	
negative	positive	03	NRAS	1	115256481	NM_002524:c.230G>A, p.Gly77Asp	
negative	negative	03	NRAS	1	115256490	NM_002524:c.221C>T, p.Thr74Ile	
negative	negative	02	NRAS	1	115256506	NM_002524:c.205G>A, p.Asp69Asn	
negative	negative	03	NRAS	1	115256508	NM_002524:c.203G>A, p.Arg68Lys	
negative	negative	07	NRAS	1	115256515	NM_002524:c.196G>A, p.Ala66Thr	
negative	negative	03	NRAS	1	115256517	NM_002524:c.194G>A, p.Ser65Asn	
negative	negative	07	NRAS	1	115256527	NM_002524:c.184G>A, p.Glu62Lys	
negative	positive	10	NRAS	1	115256527	NM_002524:c.184G>A, p.Glu62Lys	
negative	negative	05	NRAS	1	115256533	NM_002524:c.178G>A, p.Gly60Arg	rs1557982817
negative	positive	10	NRAS	1	115256542	NM_002524:c.169G>A, p.Asp57Asn	rs1465850103

Supplementary Table 5: continued

HPV-status	LDR-status	Matching pair	Gene	Chr	Position	Mutation	rsID
negative	negative	08	NRAS	1	115256562	NM_002524:c.149C>T, p.Thr50Ile	rs267606921
negative	negative	07	NRAS	1	115256569	NM_002524:c.142G>A, p.Gly48Ser	rs1444063606
negative	negative	03	NRAS	1	115256572	NM_002524:c.139G>A, p.Asp47Asn	
positive	positive	01	NOTCH1	9	139402699	NM_017617:c.3310G>A, p.Ala1104Thr	
positive	positive	02	NOTCH1	9	139402702	NM_017617:c.3307G>A, p.Val1103Met	
positive	positive	01	NOTCH1	9	139402707	NM_017617:c.3302G>A, p.Cys1101Tyr	
negative	positive	10	NOTCH1	9	139402707	NM_017617:c.3302G>A, p.Cys1101Tyr	
negative	negative	08	NOTCH1	9	139402716	NM_017617:c.3293G>A, p.Ser1098Asn	
negative	negative	08	NOTCH1	9	139402723	NM_017617:c.3286G>A, p.Val1096Met	rs369730402
negative	negative	02	NOTCH1	9	139402761	NM_017617:c.3248G>A, p.Cys1083Tyr	
negative	negative	02	NOTCH1	9	139402765	NM_017617:c.3244C>T, p.Arg1082Cys	rs1386494518
positive	positive	02	NOTCH1	9	139402773	NM_017617:c.3236C>T, p.Thr1079Ile	rs1010573276
positive	positive	02	NOTCH1	9	139402777	NM_017617:c.3232C>T, p.His1078Tyr	rs1233569898
negative	negative	08	NOTCH1	9	139402779	NM_017617:c.3230C>T, p.Thr1077Ile	
positive	positive	02	NOTCH1	9	139402785	NM_017617:c.3224G>A, p.Trp1075*	
negative	negative	09	NOTCH1	9	139412230	NM_017617:c.1415G>A, p.Gly472Glu	
negative	positive	02	NOTCH1	9	139412242	NM_017617:c.1402_1403insG, p.Leu468fs	
negative	negative	13	NOTCH1	9	139412242	NM_017617:c.1402_1403insG, p.Leu468fs	
negative	positive	11	NOTCH1	9	139412252	NM_017617:c.1393G>A, p.Ala465Thr	rs1057523819
negative	negative	08	NOTCH1	9	139412266	NM_017617:c.1379C>T, p.Pro460Leu	rs776258761
positive	positive	02	NOTCH1	9	139413099	NM_017617:c.1043C>T, p.Ala348Val	

Supplementary Table 5: continued

HPV-status	LDR-status	Matching pair	Gene	Chr	Position	Mutation	rsID
negative	positive	11	NOTCH1	9	139413120	NM_017617:c.1022G>A, p.Ser341Asn	
negative	positive	11	NOTCH1	9	139413126	NM_017617:c.1016G>A, p.Cys339Tyr	
negative	negative	03	NOTCH1	9	139413144	NM_017617:c.998G>A, p.Ser333Asn	
negative	positive	11	NOTCH1	9	139413157	NM_017617:c.985G>A, p.Gly329Ser	
negative	negative	02	NOTCH1	9	139417515	NM_017617:c.529C>T, p.Gln177*	
negative	negative	06	NOTCH1	9	139417533	NM_017617:c.511C>T, p.His171Tyr	
positive	positive	02	NOTCH1	9	139417538	NM_017617:c.506G>A, p.Ser169Asn	rs984149150
negative	negative	03	NOTCH1	9	139417541	NM_017617:c.503C>T, p.Pro168Leu	rs1330042951
negative	negative	06	NOTCH1	9	139417569	NM_017617:c.475G>A, p.Glu159Lys	rs1238603957
positive	positive	01	NOTCH1	9	139417580	NM_017617:c.464G>A, p.Cys155Tyr	
negative	negative	08	NOTCH1	9	139417617	NM_017617:c.427C>T, p.Pro143Ser	
negative	positive	11	FBXW7	4	153247264	NM_033632:c.1538G>A, p.Arg513Lys	
positive	positive	02	FBXW7	4	153247271	NM_033632:c.1531G>A, p.Gly511Ser	
negative	negative	02	FBXW7	4	153247280	NM_033632:c.1522C>T, p.Gln508*	
positive	positive	12	FBXW7	4	153247285	NM_033632:c.1517G>A, p.Cys506Tyr	
positive	positive	02	FBXW7	4	153247288	NM_033632:c.1514G>T, p.Arg505Leu	rs1057519896
negative	negative	07	FBXW7	4	153247304	NM_033632:c.1498C>T, p.His500Tyr	
negative	positive	11	FBXW7	4	153247306	NM_033632:c.1496G>A, p.Gly499Asp	
negative	negative	02	FBXW7	4	153247307	NM_033632:c.1495G>A, p.Gly499Ser	
negative	negative	03	FBXW7	4	153247308	NM_033632:c.1494G>A, p.Met498Ile	
negative	negative	06	FBXW7	4	153247316	NM_033632:c.1486G>A, p.Val496Ile	

Supplementary Table 5: continued

HPV-status	LDR-status	Matching pair	Gene	Chr	Position	Mutation	rsID
positive	positive	01	FBXW7	4	153247319	NM_033632:c.1483C>T, p.His495Tyr	
negative	negative	02	FBXW7	4	153247324	NM_033632:c.1478G>A, p.Cys493Tyr	
negative	positive	10	FBXW7	4	153247328	NM_033632:c.1474C>T, p.Gln492*	
negative	negative	03	FBXW7	4	153247333	NM_033632:c.1469C>T, p.Thr490Ile	
negative	negative	08	FBXW7	4	153247337	NM_033632:c.1465G>A, p.Glu489Lys	
positive	positive	02	FBXW7	4	153247343	NM_033632:c.1459G>A, p.Asp487Asn	
positive	positive	01	FBXW7	4	153247344	NM_033632:c.1458G>A, p.Trp486*	
positive	positive	02	FBXW7	4	153247344	NM_033632:c.1458G>A, p.Trp486*	
negative	negative	02	FBXW7	4	153247345	NM_033632:c.1457G>A, p.Trp486*	
negative	negative	06	FBXW7	4	153247349	NM_033632:c.1453G>A, p.Val485Ile	rs1325363774
negative	negative	02	FBXW7	4	153247357	NM_033632:c.1445C>T, p.Thr482Ile	
negative	negative	02	FBXW7	4	153247361	NM_033632:c.1441G>A, p.Ala481Thr	
negative	negative	02	FBXW7	4	153247366	NM_033632:c.1436G>A, p.Arg479Gln	rs866987936
positive	positive	01	FBXW7	4	153247369	NM_033632:c.1433C>T, p.Ser478Phe	
negative	negative	02	FBXW7	4	153247375	NM_033632:c.1427G>A, p.Ser476Asn	
positive	negative	03	FBXW7	4	153249370	NM_033632:c.1408C>T, p.His470Tyr	rs1169241042
negative	negative	03	FBXW7	4	153249370	NM_033632:c.1408C>T, p.His470Tyr	rs1169241042
negative	positive	10	FBXW7	4	153249376	NM_033632:c.1402C>T, p.His468Tyr	rs979863616
positive	positive	01	FBXW7	4	153249381	NM_033632:c.1397G>A, p.Cys466Tyr	
negative	positive	10	FBXW7	4	153249393	NM_033632:c.1385C>T, p.Ser462Phe	
positive	positive	02	FBXW7	4	153249403	NM_033632:c.1375G>A, p.Gly459Arg	

Supplementary Table 5: continued

HPV-status	LDR-status	Matching pair	Gene	Chr	Position	Mutation	rsID
negative	positive	10	FBXW7	4	153249403	NM_033632:c.1375G>A, p.Gly459Arg	
positive	positive	02	FBXW7	4	153249415	NM_033632:c.1363C>T, p.His455Tyr	
positive	positive	02	FBXW7	4	153249456	NM_033632:c.1322G>A, p.Arg441Gln	
negative	negative	02	FBXW7	4	153249460	NM_033632:c.1318G>A, p.Asp440Asn	
positive	positive	12	FBXW7	4	153249465	NM_033632:c.1313C>T, p.Ser438Phe	
negative	negative	08	FBXW7	4	153249469	NM_033632:c.1309G>A, p.Gly437Arg	
positive	positive	01	PIK3CA	3	178916720	NM_006218:c.107G>A, p.Cys36Tyr	
positive	positive	01	PIK3CA	3	178916731	NM_006218:c.118G>A, p.Ala40Thr	
negative	negative	13	PIK3CA	3	178916778	NM_006218:c.165_166insC, p.Tyr56fs	
negative	positive	11	PIK3CA	3	178916816	NM_006218:c.203A>G, p.Tyr68Cys	
positive	positive	02	PIK3CA	3	178916842	NM_006218:c.229G>A, p.Ala77Thr	
positive	positive	01	PIK3CA	3	178916851	NM_006218:c.238G>A, p.Glu80Lys	
negative	negative	06	PIK3CA	3	178916923	NM_006218:c.310C>T, p.Pro104Ser	
negative	positive	11	PIK3CA	3	178916924	NM_006218:c.311C>T, p.Pro104Leu	rs863225060
negative	negative	07	PIK3CA	3	178916926	NM_006218:c.313G>A, p.Val105Ile	
positive	positive	02	PIK3CA	3	178916929	NM_006218:c.316G>A, p.Gly106Ser	
positive	positive	06	PIK3CA	3	178936043	NM_006218:c.1585G>A, p.Glu529Lys	
negative	positive	02	PIK3CA	3	178936063	NM_006218:c.1606dup, p.Thr536fs	
negative	negative	13	PIK3CA	3	178936063	NM_006218:c.1606dup, p.Thr536fs	
positive	negative	03	PIK3CA	3	178936070	NM_006218:c.1612G>A, p.Asp538Asn	
positive	positive	01	PIK3CA	3	178936076	NM_006218:c.1618C>T, p.Leu540Phe	

Supplementary Table 5: continued

HPV-status	LDR-status	Matching pair	Gene	Chr	Position	Mutation	rsID
positive	negative	03	PIK3CA	3	178936082	NM_006218:c.1624G>A, p.Glu542Lys	rs121913273
positive	negative	08	PIK3CA	3	178936082	NM_006218:c.1624G>A, p.Glu542Lys	rs121913273
negative	negative	03	PIK3CA	3	178936091	NM_006218:c.1633G>C, p.Glu545Gln	rs104886003
positive	positive	06	PIK3CA	3	178936091	NM_006218:c.1633G>A, p.Glu545Lys	rs104886003
positive	positive	01	PIK3CA	3	178936113	NM_006218:c.1655G>A, p.Trp552*	
positive	positive	01	PIK3CA	3	178936114	NM_006218:c.1656G>A, p.Trp552*	
positive	positive	02	PIK3CA	3	178938812	NM_006218:c.2054G>A, p.Gly685Asp	
negative	positive	10	PIK3CA	3	178938842	NM_006218:c.2084G>A, p.Cys695Tyr	
negative	positive	03	PIK3CA	3	178952030	NM_006218:c.3085G>A, p.Asp1029Asn	
positive	positive	01	PIK3CA	3	178952054	NM_006218:c.3109G>A, p.Glu1037Lys	
negative	negative	06	PIK3CA	3	178952085	NM_006218:c.3140A>G, p.His1047Arg	rs121913279
positive	positive	12	PIK3CA	3	178952085	NM_006218:c.3140A>G, p.His1047Arg	rs121913279
negative	negative	08	PIK3CA	3	178952097	NM_006218:c.3152G>A, p.Trp1051*	
positive	positive	01	PIK3CA	3	178952110	NM_006218:c.3165G>A, p.Met1055Ile	
negative	negative	08	PIK3CA	3	178952135	NM_006218:c.3190C>T, p.Gln1064*	
negative	positive	10	BCL6	3	187447090	NM_001130845:c.1103C>T, p.Thr368Ile	rs767821014
negative	negative	06	BCL6	3	187447105	NM_001130845:c.1088C>T, p.Pro363Leu	
negative	negative	06	BCL6	3	187447106	NM_001130845:c.1087C>T, p.Pro363Ser	
negative	negative	08	BCL6	3	187447121	NM_001130845:c.1072G>A, p.Ala358Thr	
positive	positive	01	BCL6	3	187447127	NM_001130845:c.1066C>T, p.Leu356Phe	
positive	positive	01	BCL6	3	187447132	NM_001130845:c.1061G>A, p.Cys354Tyr	rs1258718328

Supplementary Table 5: continued

HPV-status	LDR-status	Matching pair	Gene	Chr	Position	Mutation	rsID
negative	negative	02	BCL6	3	187447157	NM_001130845:c.1036G>A, p.Glu346Lys	
negative	negative	03	BCL6	3	187447165	NM_001130845:c.1028C>T, p.Ser343Leu	
negative	negative	02	BCL6	3	187447175	NM_001130845:c.1018C>T, p.Gln340*	
negative	positive	03	BCL6	3	187447192	NM_001130845:c.1001C>T, p.Pro334Leu	
negative	negative	02	BCL6	3	187447327	NM_001130845:c.866G>A, p.Arg289Gln	rs776120197
negative	positive	10	BCL6	3	187447328	NM_001130845:c.865C>T, p.Arg289*	
negative	negative	08	BCL6	3	187447340	NM_001130845:c.853G>A, p.Ala285Thr	
negative	negative	08	BCL6	3	187447346	NM_001130845:c.847C>T, p.Pro283Ser	
negative	negative	08	BCL6	3	187447355	NM_001130845:c.838G>A, p.Gly280Ser	rs1210638938
negative	positive	10	BCL6	3	187447366	NM_001130845:c.827G>A, p.Ser276Asn	rs766224934
negative	negative	03	BCL6	3	187447373	NM_001130845:c.820C>T, p.His274Tyr	
negative	positive	10	BCL6	3	187447374	NM_001130845:c.819G>A, p.Met273Ile	
negative	negative	02	BCL6	3	187447379	NM_001130845:c.814G>A, p.Asp272Asn	
positive	positive	05	BCL6	3	187447384	NM_001130845:c.809G>A, p.Arg270Gln	rs780987785
negative	negative	02	BCL6	3	187447391	NM_001130845:c.802G>A, p.Glu268Lys	
negative	negative	05	BCL6	3	187447391	NM_001130845:c.802G>A, p.Glu268Lys	
positive	positive	12	BCL6	3	187447411	NM_001130845:c.782C>T, p.Pro261Leu	
positive	negative	04	BCL6	3	187447412	NM_001130845:c.781C>T, p.Pro261Ser	
positive	positive	12	BCL6	3	187447414	NM_001130845:c.779C>T, p.Ser260Leu	
negative	negative	02	FAT1	4	187540296	NM_005245:c.7444G>C, p.Val2482Leu	
negative	negative	08	FAT1	4	187540298	NM_005245:c.7442C>T, p.Thr2481Ile	

Supplementary Table 5: continued

HPV-status	LDR-status	Matching pair	Gene	Chr	Position	Mutation	rsID
negative	negative	05	FAT1	4	187540305	NM_005245:c.7435C>T, p.His2479Tyr	rs267600116
positive	positive	01	FAT1	4	187540308	NM_005245:c.7432G>A, p.Val2478Ile	
negative	negative	03	FAT1	4	187540308	NM_005245:c.7432G>A, p.Val2478Ile	
negative	positive	10	FAT1	4	187540308	NM_005245:c.7432G>A, p.Val2478Ile	
positive	positive	01	FAT1	4	187540319	NM_005245:c.7421G>A, p.Ser2474Asn	rs1422896730
positive	positive	01	FAT1	4	187540329	NM_005245:c.7411G>A, p.Val2471Ile	
negative	negative	03	FAT1	4	187540332	NM_005245:c.7408G>A, p.Gly2470Arg	
negative	negative	06	FAT1	4	187540335	NM_005245:c.7405G>A, p.Asp2469Asn	
positive	positive	02	FAT1	4	187540377	NM_005245:c.7363C>T, p.His2455Tyr	rs1258054510
positive	positive	01	FAT1	4	187540380	NM_005245:c.7360C>T, p.Arg2454Trp	rs202097333
positive	positive	02	FAT1	4	187540380	NM_005245:c.7360C>T, p.Arg2454Trp	rs202097333
negative	negative	13	FAT1	4	187540385	NM_005245:c.7354_7355insA, p.Leu2452fs	
negative	negative	03	FAT1	4	187540407	NM_005245:c.7333G>A, p.Gly2445Arg	
negative	negative	06	FAT1	4	187540407	NM_005245:c.7333G>A, p.Gly2445Arg	
positive	positive	05	FAT1	4	187630386	NM_005245:c.596C>T, p.Pro199Leu	
negative	negative	02	FAT1	4	187630400	NM_005245:c.582G>A, p.Met194Ile	
negative	negative	03	FAT1	4	187630405	NM_005245:c.577G>A, p.Asp193Asn	rs1232769040
negative	negative	02	FAT1	4	187630407	NM_005245:c.575C>T, p.Thr192Ile	
negative	negative	07	FAT1	4	187630411	NM_005245:c.571C>T, p.Arg191*	
negative	positive	07	FAT1	4	187630413	NM_005245:c.561_568del, p.Ser187fs	

Supplementary Table 5: continued

HPV-status	LDR-status	Matching pair	Gene	Chr	Position	Mutation	rsID
negative	positive	10	FAT1	4	187630414	NM_005245:c.568G>A, p.Asp190Asn	
negative	negative	02	FAT1	4	187630425	NM_005245:c.557A>C, p.Tyr186Ser	
negative	negative	07	FAT1	4	187630435	NM_005245:c.547G>A, p.Glu183Lys	
negative	negative	08	FAT1	4	187630437	NM_005245:c.545G>A, p.Gly182Glu	
negative	negative	07	FAT1	4	187630446	NM_005245:c.536G>A, p.Gly179Glu	
negative	positive	10	FAT1	4	187630473	NM_005245:c.509G>A, p.Arg170Lys	
positive	positive	01	FAT1	4	187630488	NM_005245:c.494G>A, p.Arg165Lys	rs770175041
negative	negative	13	FAT1	4	187630507	NM_005245:c.474dup, p.Pro159fs	
negative	negative	03	FAT1	4	187630516	NM_005245:c.466G>A, p.Val156Ile	rs376750393
negative	negative	08	FAT1	4	187630516	NM_005245:c.466G>A, p.Val156Ile	rs376750393
negative	negative	13	FAT1	4	187630516	NM_005245:c.465_466insT, p.Val156fs	rs376750393
negative	negative	02	FAT1	4	187630527	NM_005245:c.455C>T, p.Thr152Ile	
negative	positive	10	FAT1	4	187630552	NM_005245:c.430G>A, p.Asp144Asn	rs1211149994
positive	positive	01	FAT1	4	187630594	NM_005245:c.388G>A, p.Glu130Lys	
negative	negative	02	TP63	3	189456432	NM_001114978:c.193C>T, p.Pro65Ser	
negative	negative	03	TP63	3	189456441	NM_001114978:c.202T>C, p.Ser68Pro	
negative	negative	08	TP63	3	189456447	NM_001114978:c.208C>T, p.Gln70*	
negative	positive	10	TP63	3	189456451	NM_001114978:c.212C>T, p.Pro71Leu	
negative	negative	03	TP63	3	189456468	NM_001114978:c.229G>A, p.Val77Met	
negative	negative	02	TP63	3	189456474	NM_001114978:c.235G>A, p.Glu79Lys	rs1484040098
negative	negative	02	TP63	3	189456477	NM_001114978:c.238C>T, p.Pro80Ser	

Supplementary Table 5: continued

HPV-status	LDR-status	Matching pair	Gene	Chr	Position	Mutation	rsID
positive	positive	02	TP63	3	189456478	NM_001114978:c.239C>T, p.Pro80Leu	
negative	negative	06	TP63	3	189456483	NM_001114978:c.244G>A, p.Glu82Lys	
negative	negative	03	TP63	3	189456489	NM_001114978:c.250G>A, p.Gly84Ser	
positive	positive	12	TP63	3	189526233	NM_001114978:c.497C>T, p.Pro166Leu	
negative	negative	02	TP63	3	189526242	NM_001114978:c.506C>T, p.Thr169Ile	
negative	negative	03	TP63	3	189526251	NM_001114978:c.515C>A, p.Pro172Gln	
negative	negative	05	TP63	3	189526251	NM_001114978:c.515C>T, p.Pro172Leu	
positive	positive	02	TP63	3	189526257	NM_001114978:c.521C>T, p.Pro174Leu	rs201188464
positive	positive	02	TP63	3	189526287	NM_001114978:c.551C>T, p.Ser184Leu	
positive	positive	01	TP63	3	189526304	NM_001114978:c.568G>A, p.Ala190Thr	
negative	negative	06	TP63	3	189526305	NM_001114978:c.569C>T, p.Ala190Val	
negative	negative	03	TP63	3	189526311	NM_001114978:c.575G>A, p.Trp192*	
negative	positive	02	TP63	3	189585622	NM_001114978:c.883G>A, p.Val295Ile	
negative	positive	06	TP63	3	189585631	NM_001114978:c.894del, p.Glu298fs	
negative	negative	06	TP63	3	189585631	NM_001114978:c.894del, p.Glu298fs	
negative	negative	07	TP63	3	189585631	NM_001114978:c.894del, p.Glu298fs	
negative	negative	08	TP63	3	189585631	NM_001114978:c.892G>A, p.Glu298Lys	
negative	negative	08	TP63	3	189585638	NM_001114978:c.899C>T, p.Thr300Met	rs886058222
negative	negative	03	TP63	3	189585662	NM_001114978:c.923G>A, p.Cys308Tyr	
negative	negative	02	TP63	3	189585668	NM_001114978:c.929G>A, p.Ser310Asn	
negative	negative	02	TP63	3	189585683	NM_001114978:c.944G>A, p.Gly315Glu	

Supplementary Table 5: continued

HPV-status	LDR-status	Matching pair	Gene	Chr	Position	Mutation	rsID
negative	positive	10	TP63	3	189585694	NM_001114978:c.955C>T, p.Arg319Cys	rs121908839
negative	positive	02	TP63	3	189586396	NM_001114978:c.1020T>G, p.Phe340Leu	
negative	negative	13	TP63	3	189586396	NM_001114978:c.1020T>G, p.Phe340Leu/	
negative	positive	11	TP63	3	189586400	NM_001114978:c.1024G>A, p.Ala342Thr	
negative	negative	03	TP63	3	189586401	NM_001114978:c.1025C>T, p.Ala342Val	
negative	positive	11	TP63	3	189586418	NM_001114978:c.1042C>T, p.Pro348Ser	
positive	positive	02	TP63	3	189586445	NM_001114978:c.1069G>A, p.Asp357Asn	
positive	positive	11	TP63	3	189586479	NM_001114978:c.1106dup, p.Asn370fs	
positive	positive	13	TP63	3	189586479	NM_001114978:c.1106dup, p.Asn370fs	
negative	positive	11	TP63	3	189587139	NM_001114978:c.1156C>T, p.Gln386*	
positive	positive	02	TP63	3	189587146	NM_001114978:c.1163C>T, p.Thr388Ile	
positive	positive	02	TP63	3	189587161	NM_001114978:c.1178G>A, p.Arg393Gln	rs1173679499
negative	positive	10	TP63	3	189608604	NM_001114978:c.1679C>T, p.Ser560Leu	
negative	negative	06	TP63	3	189608612	NM_001114978:c.1687G>A, p.Asp563Asn	
negative	positive	10	TP63	3	189608612	NM_001114978:c.1687G>A, p.Asp563Asn	
positive	positive	01	TP63	3	189608630	NM_001114978:c.1687G>A, p.Gly569Arg	
positive	positive	01	TP63	3	189608640	NM_001114978:c.1715C>T, p.Thr572Ile	
negative	negative	05	TP63	3	189608664	NM_001114978:c.1739C>T, p.Ser580Phe	rs1464459460
negative	negative	08	TP63	3	189608669	NM_001114978:c.1744G>A, p.Asp582Asn	rs865905084

LDR:local/distant recurrence; Chr:Chromosome
