# Supplementary material for: Mutation patterns in recurrent and/or metastatic oropharyngeal squamous cell carcinomas in relation to human papillomavirus status
Source: Cancer Med. 2021 Feb 1;10(4):1347–56. doi: 10.1002/cam4.3741 (PMC7926014; doi:10.1002/cam4.3741)
Supplement: Supplementary file 7 — Table S6 [file CAM4-10-1347-s007.pdf]

Supplementary Table 6: P-values of the mutation frequencies between HPV+ OPSCC of patients with LDR and HPV- OPSCC.

**HPV+ OPSCC of patients with LDR**	**HPV- OPSCC**		
	**all**	**patients without LDR**	**patients with LDR**
TP53	**0.014**	**0.017**	0.059
RB1	0.179	0.078	0.549
STK11	0.653	0.931	0.486
CDH1	0.492	0.274	1.000
HRAS	0.260	0.739	0.102
KRAS	0.065	**0.034**	0.150
NRAS	0.155	0.063	0.521
FAT1	0.461	0.113	0.606
PIK3CA	0.935	0.890	0.773
PIK3R1	0.973	0.419	0.370
PTEN	0.805	0.387	0.521
FANCA	0.639	0.404	0.940
FBXW7	0.900	0.507	0.582
CYLD	0.334	0.273	0.521
BCL6	0.707	0.326	0.655
TP63	0.646	0.307	0.781
TAF1	0.809	0.677	0.353
EP300	0.886	0.525	0.630
DDX3X	0.445	0.217	0.954
NOTCH1	0.325	0.175	0.731
JAK1	0.694	0.521	1.000
JAK2	0.770	0.650	0.257
PDGFRA	0.546	0.353	0.940

P-values were calculated by Mann-Whitney-U test for independent samples; p-values ≤0.05 in bold.
